# Small Molecules Derived from Thieno[3,4-*c*]pyrrole-4,6-dione (TPD) and Their Use in Solution Processed Organic Solar Cells

**DOI:** 10.3390/molecules22101607

**Published:** 2017-09-30

**Authors:** Cesar Garcias-Morales, Daniel Romero-Borja, José-Luis Maldonado, Arián E. Roa, Mario Rodríguez, J. Pablo García-Merinos, Armando Ariza-Castolo

**Affiliations:** 1 Research Group of Optical Properties of Materials (GPOM), Centro de Investigaciones en Óptica, A.P. 1-948, 37000 León, Guanajuato, Mexico; ad.romero.borja@cio.mx (D.R.-B.); espinosa.arian@gmail.com (A.E.R.); mrodri@cio.mx (M.R.); 2 Instituto de Investigaciones Químico Biológicas Universidad Michoacana de San Nicolás de Hidalgo Edificio B-1. Ciudad Universitaria, 58030 Morelia, Michoacán, Mexico; jpgarciam@gmail.com; 3 Departamento de Química, Centro de Investigación y de Estudios Avanzados del Instituto Politécnico Nacional, Avenida Instituto Politécnico Nacional 2508 Colonia San Pedro Zacatenco, 07360 Mexico, D.F., Mexico; aariza@cinvestav.mx

**Keywords:** organic small molecules, microwave synthesis, donor-acceptor-donor structure, organic solar cells

## Abstract

In this work, microwave synthesis, chemical, optical and electrochemical characterization of three small organic molecules, **TPA-TPD**, **TPA-PT-TPD** and **TPA-TT-TPD** with donor-acceptor structure and their use in organic photovoltaic cells are reported. For the synthesis, 5-(2-ethylhexyl)-4*H*-thieno[3,4-*c*]pyrrole-4,6(5*H*)-dione was used as electron withdrawing fragment while the triphenylamine was used as electron donating fragment. Molecular electronic geometry and electronic distribution density were established by density functional theory (DFT) calculations and confirmed by optical and chemical characterization. These molecules were employed as electron-donors in the active layer for manufacturing bulk heterojunction organic solar cells, where [6,6]-phenyl C71 butyric acid methyl ester (PC71BM) was used as electron-acceptor. As cathode, Field′s metal (FM), an eutectic alloy (Bi/In/Sn: 32.5%, 51%, and 16.5%, respectively) with a melting point above 62 °C, was easily deposited by drop casting under vacuum-free process and at air atmosphere. Prepared devices based on **TPA-TPD**:PC71BM (1:4 *w*/*w* ratio) presented a large V_OC_ = 0.97 V, with J_SC_ = 7.9 mA/cm^2^, a FF = 0.34, then, a power conversion efficiency (PCE) of 2.6%.

## 1. Introduction

The synthesis and study of organic semiconducting molecules have increased considerably in recent years due to their electronic and optoelectronic properties [1,2,3,4,5]; it is because their chemical structure can be easily modified, resulting in a large number of organic molecules with tunable electronic and optoelectronic characteristics. Due to these optical and electrical properties, organic molecules are being used in the manufacture of organic solar cells (OSCs) [6,7,8,9], organic thin film transistors (OFETs) [10,11,12] and organic light emitting diodes (OLEDs) [13,14,15]. 

Particularly, OSCs have attracted great attention because they are a source of renewable energy and their manufacture is inexpensive, they are thin, lightweight as well as semi-transparent; further, because of their potential flexibility, these devices can be used almost on any surface [16,17,18,19,20]. The key in the development of OSCs is the large number in the design and synthesis of organic semiconductor materials that allow high power conversion efficiency, mainly polymers and small molecules (SMs) [21]. SMs have certain advantages with respect to polymers because they have defined molecular weights, good solubility, high reproducibility in each batch of synthesis, as well as an easier process of purification and characterization compared with polymers [6,22,23]. 

The synthesis strategy of SMs for OSCs applications has focused mainly on molecules where an electron donating fragment (donor D) is covalently bounded to an electron withdrawing fragment (acceptor A) obtaining a Donor-Acceptor structure. This structure helps to modulate the energy difference between the highest occupied molecular orbital (HOMO) and lowest unoccupied molecular orbital (LUMO) in order to obtain high open circuit voltage (V_OC_) values in photovoltaic devices [24,25,26,27]. There are a large variety of acceptor fragments used in the synthesis of SMs; one of them is 5-(2-ethylhexyl)-4*H*-thieno[3,4-*c*]pyrrole-4,6(5*H*)-dione (TPD), which is attractive because it is symmetric, compact and rigid [28,29]. Organic molecules based on TPD rings give coplanar and rigid structures and also form a quinoid thiophene-maleimide arrangement in order to stabilize the structure and the excited energy state [30,31]. TPD fragment is used mainly in polymer synthesis, where power conversion efficiencies up to 7% have been reported [32]. Nevertheless, the use of TPD in the synthesis of SMs has been limited to some molecules in comparison with derivatives from 2,1,3-Benzothiadiazole (BT), Isoindigo (iI) and diketo pyrrolo pyrrole (DPP) rings [33,34]. 

In OSCs, the use of TPD allows to reach high values of V_OC_ because the HOMO energy level is controlled precisely by the TPD fragment, with HOMO values below −5.2 eV [35], and high values of charge mobility: up to µ_h_ = 0.03 cm^2^ V^−1^ s^−1^ [36]. These properties make TPD an attractive acceptor fragment for the design and synthesis of molecules for OSCs applications. For instance, Liu X. et al. [37] reported the synthesis of intermediate-sized molecules using TPD as electron withdrawing fragment, which were used in the manufacture of OSCs reaching a power conversion efficiency (PCE) of 4.0%. Likewise, Lin Y. et al. [38] reported OSCs based on TPD small molecules with PCE of 3.31%. Choi Y. S. et al. [36] reported OSCs based on DTS(HexTPD2T)_2_ small molecule with PCE of 6.0%. 

On the other hand, as a strategy for the SMs synthesis, triphenylamine (TPA) is used because it is a good electron donating molecule [39], then, together with TPD as electron withdrawing fragment, molecules under the donor-acceptor structure are obtained. Organic molecules with this structure have moderate charge mobility [40]. Thus, in this work the synthesis, chemical, optical and electrochemical characterization of three molecular derivatives from TPD: **TPA-TPD**, **TPA-PT-TPD** and **TPA-TT-TPD** besides their use in the manufacture of OSCs, are reported. Synthesis was carried out by microwave assistance, which allows decreasing the reaction times for Suzuki coupling reactions and direct arylation. Chemical structure characterization was performed by IR, mass spectrometry, NMR of ^1^H and ^13^C; optical characterization was performed by UV spectroscopy. HOMO and LUMO energy levels were determined by cyclic voltammetry and by theoretical calculations using density functional theory (DFT). Organic solar cells were fabricated under the bulk heterojunction (BHJ) approach with the ITO/PEDOT:PSS/SM:PC71BM/PFN/FM architecture. FM = Field′s metal is an eutectic alloy (Bi/In/Sn: 32.5%, 51%, and 16.5%, respectively) with a melting point above 62 °C, which has been used in our research group as cathode for the fabrication of OSCs reaching power efficiencies about 2–3% based on P3HT and 7% based on PTB7 [41,42,43]. OSCs based on **TPA-TPD**:PC71BM (1:4 D:A *w*/*w* ratio) presented high V_OC_ values (~1 V) due to the low HOMO energy levels and a PCE as high as 2.6% with J_SC_ = 7.9 mA/cm^2^ and FF = 0.34. 

## 2. Results and Discussion

### 2.1. Synthesis and Characterization

**Scheme 1:**
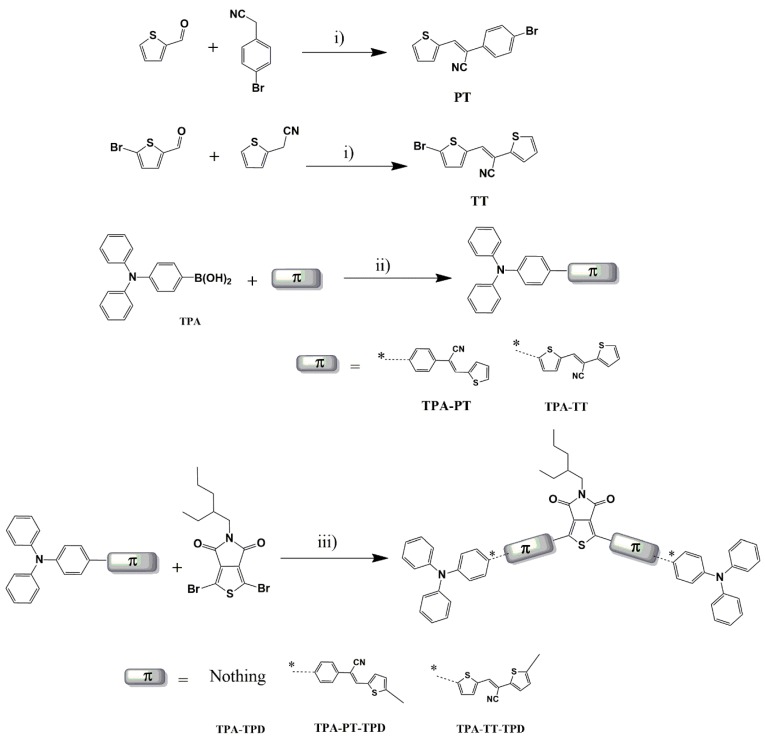
Synthetic route of **TPA-TPD**, **TPA-PT-TPD** and **TPA-TT-TPD**. (i) KOH aq 10%, EtOH, 30 min, rt; (ii) potassium pivalate (PIVOK), Pd(PPh_3_)_4_ 5% mol, dimethylacetamide (DMA), 110 °C 1 h, MW; (iii) PIVOK, Pd(OAc)_2_ 5% mol, DMA, 110 °C 1 h, MW.

Synthetic route of the conjugated organic small molecules (SMs) are depicted in Scheme 1. Chang, S.Y. et al. [44] reported the 1,3-bis(4-(diphenylamino)phenyl)-5-(2-ethylhexyl)-4*H*-thieno[3,4-*c*]pyrrole-4,6(5*H*)-dione (**TPA-TPD**) synthesis by direct arylation under conventional heating at 110 °C for 48 h using Pd(OAc)_2_ as catalyst, reaching yields of 51%. In this work, the synthesis of **TPA-TPD** is reported through Suzuki reaction by using Pd(PPh_3_)_4_ as catalyst and microwave (MW) heating at 110 °C for 1 h, getting yields of 70%. For the synthesis of (2*Z*,2′*Z*)-3,3′-(5,5′-(5-(2-ethylpentyl)-4,6-dioxo-5,6-dihydro-4*H*-thieno-[3,4-*c*]pyrrole-1,3-diyl)bis(thiophene-5,2-diyl))bis(2-(4′-(diphenylamino)-[1,1′-biphenyl]-4-yl)acrylonitrile) (**TPA-PT-TPD**) and (2*E*,2′*E*)-2,2′-(5,5′-(5-(2-ethylpentyl)-4,6-dioxo-5,6-dihydro-4*H*-thieno[3,4-*c*]pyrrole-1,3-diyl)bis(thiophene-5,2-diyl))bis(3-(5-(4-(diphenylamino)phenyl)thiophen-2-yl)acrylonitrile) (**TPA-TT-TPD**), firstly the synthesis of (*Z*)-2-(4-bromophenyl)-3-(thiophen-2-yl)acrylonitrile (PT) was carried out by Knoevenagel condensation between 4-Bromophenylacetonitrile and 2-thiophenecarboxaldehyde, while the synthesis of (*E*)-3-(5-bromothiophen-2-yl)-2-(thiophen-2-yl)acrylonitrile (TT) was carried out by Knoevenagel reaction between 2-thienylacetonitrile and 5-Bromo-2-thiophenecarboxaldehyde. 

Subsequently, through Suzuki reaction between 4-(*N*,*N*-diphenylamine)-1-phenylboronic acid with PT or TT precursors, using Pd(PPh_3_)_4_ as catalyst and microwave heating, (*Z*)-2-(4′-(diphenylamino)-[1,1′-biphenyl]-4-yl)-3-(thiophen-2-yl)acrylonitrile (TPA-PT) and (*E*)-3-(5-(4-(diphenylamino)phenyl)thiophen-2-yl)-2-(thiophen-2-yl)acrylonitrile (TPA-TT) were obtained; finally **TPA-PT-TPD** and **TPA-TT-TPD** were synthesized through direct arylation reaction between TPA-PT and TPA-TT with 1,3-dibromo-5-(2-ethylhexyl)-4*H*-thieno[3,4-*c*]pyrrole-4,6(5*H*)-dione (TPD), using Pd(OAc)_2_ as catalyst and microwave heating by 1 h.

It is important to highlight the use of microwaves because they make the synthesis cheaper than the conventional heating method. Furthermore, the microwave reaction consumes less time of synthesis; in our case, the reaction time was reduced from 48 to 1 h [44,45,46]. In addition, it produces higher reactions yields. For these mentioned reasons, microwave assistance is considered a viable method for the synthesis of organic molecules for optoelectronic applications. The chemical structures of precursors as well as those for SMs were determined by infrared (IR), mass-spectrometry and ^1^H and ^13^C.

### 2.2. Theoretical Calculations

**Figure 1:**
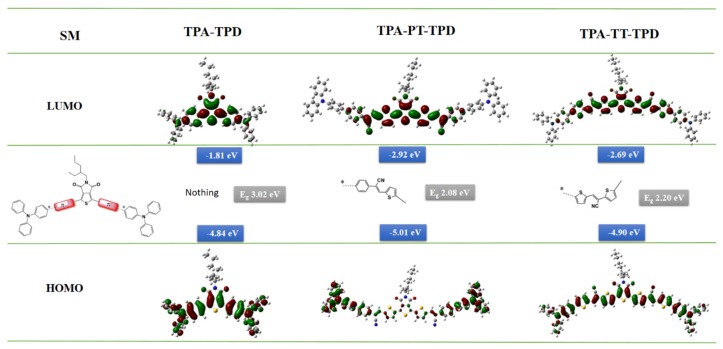
Optimized geometries, electron-state-density distributions and highest occupied molecular orbital-lowest unoccupied molecular orbital (HOMO-LUMO) energy levels of **TPA-TPD**, **TPA-PT-TPD** and **TPA-TT-TPD** using density functional theory (DFT) at the B3LYP/6-31G (d) level.

Geometrical structure and density electronic properties of the reported SMs were studied by theoretical calculation using density functional theory (DFT) with Gaussian 09 package [47]. Geometrical structures of the three molecules in their ground state were optimized without any restriction at the B3LYP/6-31G(d) level of theory [48]. The fully optimized structures were further analyzed by harmonic vibrational frequency to ensure that a real global minimum was found without imaginary vibrational frequencies. Figure 1 shows the minimum-energy geometrical conformations and the molecular orbital electron state-density distributions for HOMO and LUMO levels. HOMO-LUMO energy levels and theoretical band-gap (E_g_^cal^) calculated for these SMs are also shown in Figure 1. 

Figure 1 shows the electronic density distribution of **TPA-TPD** and **TPA-TT-TPD**. It is observed that electronic density in the HOMO orbital is delocalized along the π-conjugated system for these molecules, whereas for **TPA-PT-TPD** it is located in the TPA fragment. It is because the geometry of **TPA-TPD** and **TPA-TT-TPD** is flat, which allows the delocalization of the electrons. The geometry of **TPA-PT-TPD** is not flat, so the orbitals are not aligned for an electronic delocalization. On the other hand, in the LUMO orbital, the electronic density in the three molecules is located on the TPD fragment. It is because TPA groups act as donor groups of the electronic density, while TPD molecules act as an acceptor of the electronic density, so we conclude that electronic architecture for the three SMs is D-A-D.

The molecule with the highest value of E_g_^cal^ is **TPA-TPD** with 3.02 eV, while **TPA-PT-TPD** has the lowest one: 2.08 eV; this fact agrees with the size of the π-conjugated system. However, the value of E_g_^cal^ for **TPA-TT-TPD** (2.20 eV) is reversed in comparison with the experimental data (see below). This fact could be because, in solid state, the crystal packing plays a very important role in the optical properties due to stronger intermolecular chemical interactions [49]. However, in theoretical calculations, molecule crystal packing was not considered. 

### 2.3. Optical Properties

**Figure 2:**
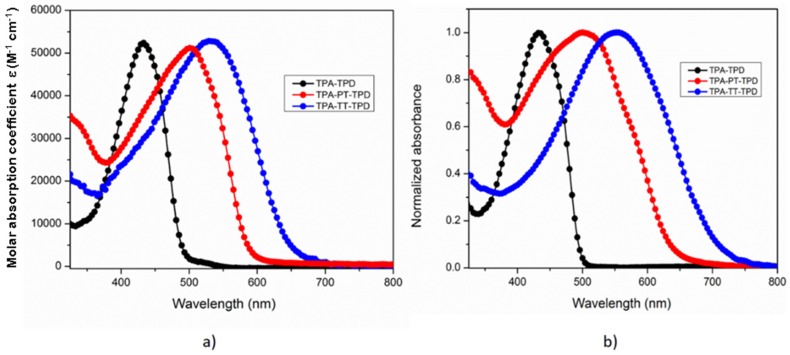
Ultraviolet–visible (UV-Vis) absorption spectra of **TPA-TPD**, **TPA-PT-TPD** and **TPA-TT-TPD** (**a**) In solution (CHCl_3_); (**b**) In solid-state films.

Figure 2 displays the molar absorption coefficient of the SMs in solution (Figure 2a), as well as the normalized ultraviolet–visible (UV-Vis) absorption spectra in solid thin films (Figure 2b). Molar absorption coefficients of **TPA-TPD**, **TPA-PT-TPD** and **TPA-TT-TPD** are very similar: 52,382, 51,200 and 52,892 M^−1^ cm^−1^ respectively. 

Table 1 summarizes the λ_max_ absorption values, the optical band-gap (E_g_^opt^) and molar absorption coefficient (ε_max_). The maximum absorption band of the molecules in solution is according to the length of the π-conjugation system. **TPA-TPD** molecule has the shorter conjugation system with λ_max_ in 433 nm, for **TPA-PT-TPD** the λ_max_ is shown at 495 nm and for **TPA-TT-TPD** the absorption was shifted towards the red region with λ_max_ at 527 nm. For **TPA-TPD** and **TPA-PT-TPD**, the difference between λ_max_ absorption in solution and λ_max_ absorption in thin solid films is less than 5 nm; this suggests that, in solution, the molecules have a rigid structure. Also, the absorption bands in thin-films are wider than those in solution; it is attributed to the molecule rigidity which favors the co-planarity and therefore a better packing of the molecule through the π-π stacking. For **TPA-TT-TPD**, the absorption shift in solid state was 24 nm towards the red zone in comparison with that in solution. It suggests that **TPA-TT-TPD** has a quinoid form in the solid state or crystalline packaging promotes the π-π stacking, so the E_g_^opt^ is reduced [38]. In addition, E_g_^opt^ was determined from the onset absorption bands of thin film spectra, values of E_g_^opt^ for **TPA-TPD**, **TPA-PT-TPD** and **TPA-TT-TPD** are: 2.48 eV, 1.90 eV, and 1.74 eV, respectively. 

### 2.4. Electrochemical Properties

**Figure 3:**
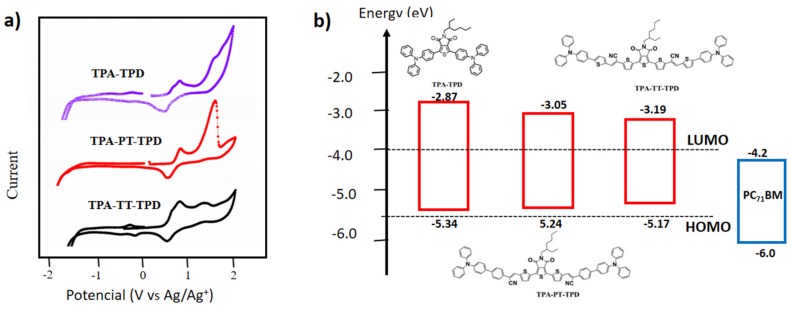
(**a**) Cyclic voltammogram (CV) (50 mV s^−1^) and (**b**) HOMO-LUMO electrochemical energy-level diagrams of **TPA-TPD**, **TPA-PT-TPD** and **TPA-TT-TPD**.

SMs were evaluated by cyclic voltammetry (CV). The recorded CV curves can be shown in Figure 3 and extracted data are listed in Table 1. For **TPA-TPD**, **TPA-PT-TPD** and **TPA-TT-TPD**, the onset oxidation potentials ($E_{onset}^{ox}$) are determined to be 0.68, 0.62 and 0.55 V, respectively. The HOMO energy for the SMs was calculated based on the equation *E_HOMO_* = −[(*E_ox_* + 4.4) + 0.22] eV, where *E_ox_* is the recorded onset oxidation potentials of SMs. 4.4 eV is the energy level of ferrocene/ferrocenium (Fc/Fc^+^) below the vacuum level and 0.22 eV is the correction by the reference used electrode [50]. The resulting HOMO values were −5.34, −5.24 and −5.17 eV, for **TPA-TPD**, **TPA-PT-TPD** and **TPA-TT-TPD**, respectively. LUMO energy levels were determined using the onset reduction potentials to be −2.47, −3.05 and −3.19 eV for **TPA-TPD**, **TPA-PT-TPD** and **TPA-TT-TPD**, respectively. 

Figure 3b shows the comparison of the HOMO-LUMO energies for the three molecules with respect to those for PC71BM, which is used as an electron acceptor material in OSCs. In addition, the energy levels of an ideal molecule (dashed lines) are shown. The energy difference between the HOMO of the molecule and the LUMO of the PC71BM is one of the main factors that determines the cell V_OC_ value. 

According to the HOMO-LUMO energy levels, it is expected that the **TPA-TPD** molecule has the highest V_OC_ value among the three SMs when it is used as donor in the active layer of a solar cell (from the theoretical point of view, V_OC_ should be 1.14 V if PC71BM is used as electron acceptor) [27], while the **TPA-TT-TPD** molecule will present a lower value of V_OC_ (1.04 V). 

On the other hand, for a proper exciton dissociation, it is required a minimum of 0.3 eV difference between the LUMO of the donor molecule and the LUMO of the PC71BM. The three SM molecules fulfill this condition, so they are candidates for using them in the manufacture of OSCs.

### 2.5. Photovoltaic Performance

**Figure 4:**
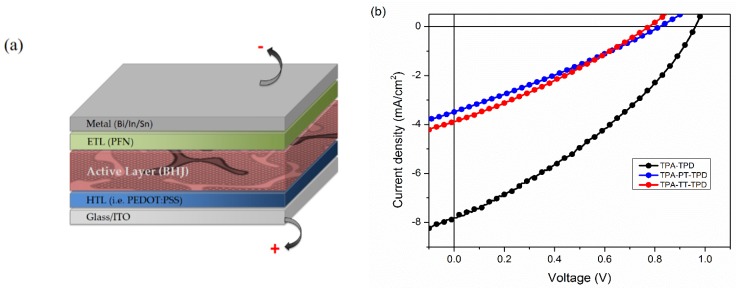
(**a**) Organic solar cells (OSCs) with the ITO/PEDOT:PSS/SM:PC71BM/PFN/FM architecture; (**b**) Current density-voltage characteristics of OSC devices based on **TPA-TPD**, **TPA-PT-TPD** and **TPA-TT-TPD**.

OSCs were fabricated under the BHJ approach by using **TPA-TPD**, **TPA-PT-TPD** and **TPA-TT-TPD** as electron donors and PC71BM as the electron acceptor in the active layer. The generally-used configuration for the devices was ITO/PEDOT:PSS/SM:PC71BM/PFN/FM (Figure 4a). The active layer was spin coated from a chlorobenzene solution of SM:PC71BM; ratios were varied from 1:1 to 1:4 *w*/*w* D:A (see Supplementary Materials: SM). The average PCE was determined from eight cells manufactured under the same conditions (see SM).

Figure 4b displays the J-V curves, and the photovoltaic parameters values are summarized in Table 2. Under the optimized conditions, the best photovoltaic parameters were reached in OSCs based on **TPA-TPD**:PC71BM (1:4 *w*/*w* D:A), with a power conversion efficiency of 2.6%, J_SC_ = 7.9 mA/cm^2^, V_OC_ = 0.97 V, and FF = 0.33. On the other hand, the photovoltaic parameters for OSCs based on **TPA-PT-TPD** and **TPA-TT-TPD** showed smaller values with PCE of 0.79%, J_SC_ = 3.5 mA/cm^2^, V_OC_ = 0.82 V and FF = 0.28 for **TPA-PT-TPD**, and PCE of 0.91%, J_SC_ = 3.9 mA/cm^2^, V_OC_ = 0.79 V, FF = 0.29 for **TPA-TT-TPD**. These measured high values of V_OC_ are consistent with those reported for OSCs based on molecules containing TPD ring as electron withdrawing. 

For instance, Lin, Y. et al. [38] reported cells with the architecture: ITO/PEDOT:PSS/SM:PC71BM/Al, getting V_OC_ of 0.94 V, while Kim, Y.J. et al. [51] reported devices with V_OC_ of 0.92 V, under the architecture: ITO/PEDOT:PSS/BDTSe-dTTP:PC71BM/LiF/Al. Short circuit current densities J_SC_ reported for OSC using SMs with TPD fragment by Lin, Y. et al. [38] are in the range of 6–8 mA/cm^2^, while the current density reached for cells manufactured with **TPA-TPD**:PC71BM in this work is 6.7 mA/cm^2^ on average (best one = 7.9 mA/cm^2^). Nevertheless, PCEs (2.6%) are still lower compared to those reported by Liu X. [37] and Lin Y. [38] (4.0% and 3.31%, respectively). This fact is mainly due to the low fill factor reached for our devices (see discussion on the next section). Another probable reason of the better PV performance for cells based on **TPA-TPD** compound could be having a larger charge mobility value than the other two SMs. For the **TPA-PT-TPD** and **TPA-TT-TPD** chemical structures, nitriles groups, which are electro-acceptors, were introduced. Additionally, the exocyclic double bonds are also presented. These features could causes changes in the electronic mobility due to variations in the molecular crystalline packaging [52,53]. 

### 2.6. Morphological Analysis

**Figure 5:**
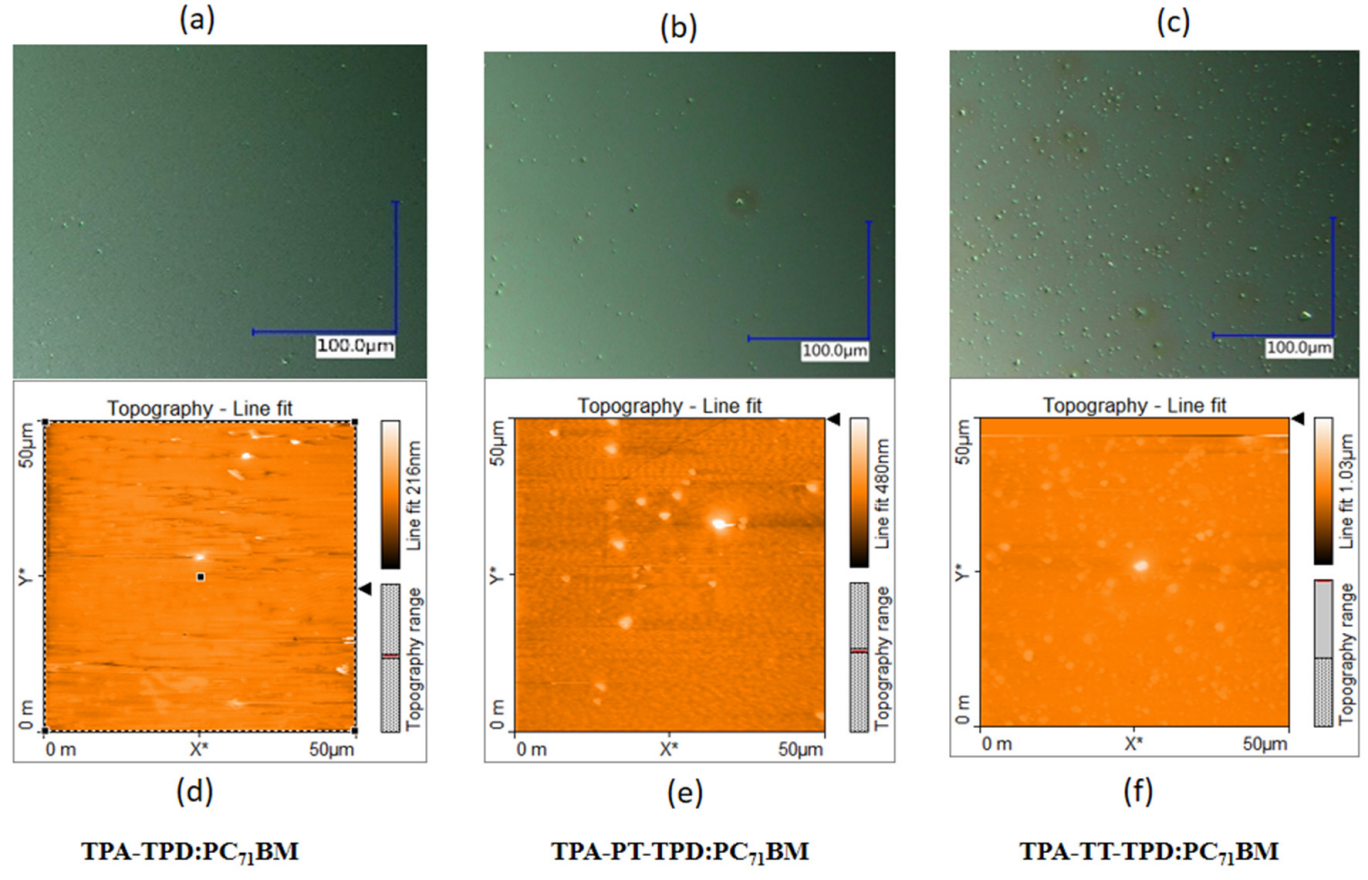
Images of SMs:PC71BM blended films prepared from CB onto ITO/PEDOT:PSS, with thermal annealing of 80 °C for 20 min, rations 1:4, 1:3 and 1:2 Donor-Acceptor for **TPA-TPD**, **TPA-PT-TPD** and **TPA-TT-TPD**, respectively: (**a**–**c**) are images from an optical microscope; (**d**–**f**) morphology images from atomic force microscopy AFM.

Figure 5a–c shows images of the active layer of SM:PC71BM obtained with an optical microscope. At this micro-scale, it is observed that films prepared with the blends **TPA-PT-TPD**:PC71BM and **TPA-TT-TPD**:PC71BM exhibit some irregularities as large as 1 µm with respect to those based on **TPA-TPD**:PC71BM, which is attributed to aggregates.

Figure 5d–f shows the surface morphology images for active layers acquired by atomic force microscopy AFM. **TPA-TPD**:PC71BM film blend exhibited a relatively flat surface with a root-mean-square (RMS) roughness of 10 nm, while for films based on **TPA-PT-TPD**:PC71BM and **TPA-TT-TPD**:PC71BM, RMS roughness values are 22 and 28 nm, respectively. These morphology irregularities could be since **TPA-PT-TPD** and **TPA-TT-TPD** are not completely miscible with the PC71BM (in these two cases, a smaller amount of the acceptor was used; see Table 2). Then, in Figure 5c,f (and to a lesser extent in Figure 5b,e) aggregates can be clearly observed. These micro-aggregates can provoke charge recombination, which causes a decrease in the current density values. Further, these micro-aggregates could also act as traps that increase the overall device resistance, which gives rise to low FF values (0.27–0.33) [54,55,56,57]. 

On the other hand, the use of TPD ring can also be compared with the use of DPP and iI as electron withdrawing fragment, both linked to the same electron donating molecule (TPA), for instance, **TPA-DPP** and **iI(TPA)_2_** molecules [33,34].

Table 3 shows this photovoltaic comparison. The architecture for each cell is: ITO/PEDOT:PSS/**TPA-DPP**:PC71BM/Al, ITO/PEDOT:PSS/**iI(TPA)_2_**:PC61BM/Ca/Al and ITO/PEDOT:PSS/**TPA-TPD**:PC71BM/PFN/FM (this work). OSCs based on TPD ring have the largest J_SC_ value (7.9 mA/cm^2^) and the highest V_OC_ value (0.97 V), therefore owing a higher PCE (2.6% compared to 1.0 and 0.84% for **TPA-DPP** and **iI(TPA)_2_**, respectively. Among the three molecules, the HOMO energy level of **TPA-TPD** is the closest to the energy level of the ideal molecule; for this reason it presents the highest value of V_OC_ in comparison with the other two molecules.

**Figure 6:**
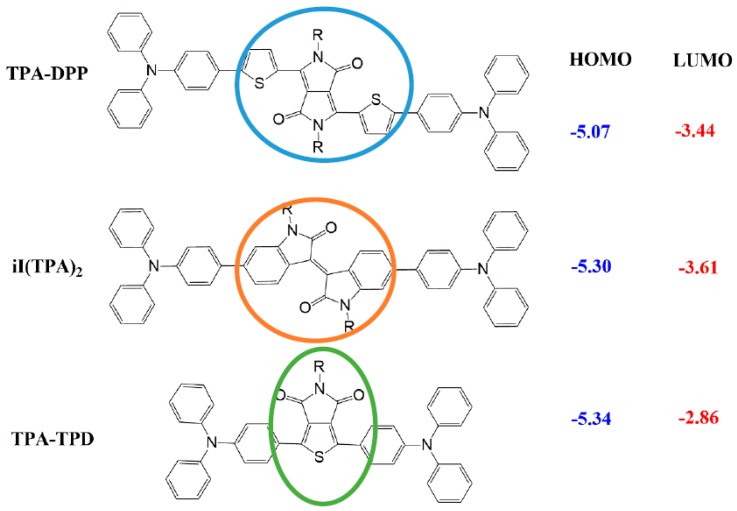
HOMO-LUMO energy level comparison of two low molecular weight molecules with D-A-D structures using **iI** [33], **DPP** [34], with respect to **TPD** as A fragments.

The HOMO-LUMO energy levels for **TPA-DDP**, **iI(TPA)_2_** and **TPA-TPD** are shown in Figure 6, where **TPA-DDP** has the lower E_g_ value of 1.63 eV, followed by **iI(TPA)_2_** and **TPA-TPD** with 1.69 eV and 2.48 eV, respectively. According to these data, OSCs based on **TPA-DDP** and **iI(TPA)_2_** should have better efficiencies than OSCs based on **TPA-TPD**; however, the opposite was observed. To elucidate this behavior, it is necessary to not only analyze orbitals energies, but also geometries determination, as well as their electronic distribution analysis, among other inherent facts related to the film formation.

## 3. Materials and Methods

### 3.1. Materials

Potassium hydroxide was purchased from J.T. Baker (León, GTO., Mexico), 4-bromophenylacetonitrile was obtained from Alfa Aesar. Tetrakis(triphenylphosphine)palladium (Pd(PPh_3_)_4_), palladium (II) acetate (Pd(OAc)_2_), 4-(*N*,*N*-diphenylamine)-1-phenylboronic acid, 4-bromotriphenylamine, 2,2-dimethylpropionic acid, 2-thienylacetonitrile, 5-bromo-2-thiophenecarboxaldehyde, 2-thiophenecarboxaldehyde and 1,3-dibromo-5-(2-ethylhexyl)-4*H*-thieno[3,4-*c*]pyrrole-4,6(5*H*)-dione, were purchased from Sigma-Aldrich (Toluca, Mexico). The other reagents and solvents were purchased from Karal (León, GTO., Mexico). All reagents and chemicals were used without further purification unless stated otherwise. The potassium pivalate (PIVOK) was synthesized according to the literature [58].

### 3.2. Characterization

Fourier transform infrared (FTIR) spectra were acquired using a Varian 640 spectrophotometer (Varian now Agilent Technologies Mexico, Mexico City) by the ATR method. The absorption spectra were measured using a PerkinElmer/Lambda 900 model UV/VIS/NIR spectrophotometer (PerkinElmer, Mexico), for solution spectra SMs were dissolved in chloroform, while in solid thin film SMs were deposited on glass substrates by spin-coating; ^1^H and ^13^C-NMR spectra were recorded at 21 ± 1 °C using a JEOL ECA 500 MHz spectrometer (JEOL, Mexico City). Spectra were recorded in CDCl_3_ solution, chemical shifts were referenced to tetramethylsilane (CH_3_)_4_Si, which was used as an internal standard (δ ^1^H = 0, δ ^13^C = 0). Mass spectra were recorded in an Agilent G1969 LC/MSD TOF spectrometer (Agilent Technologies Mexico, Mexico) coupled to HPLC with electrospray ionization. 

### 3.3. Synthesis

#### 3.3.1. Knoevenagel Reaction

In a 50 mL flask, the aryl-acetonitrile and the corresponding aldehyde are placed and then 25 mL of ethanol is added. The mixture is stirred for 5 min, then 5 drops of an aqueous potassium hydroxide solution at 10% is added to the mixture, the reaction is stirred and heated up to 50 °C, it was monitored by thin layer chromatography until the reaction is complete, then the mixture is cooled down to 0 °C, the formation of a crystalline precipitate is observed, it is filtered and washed using cold EtOH. The solid is recovered and purified by recrystallization by using a solvent mixture of acetone/EtOH 40:60.

*(Z)-2-(4-Bromophenyl)-3-(thiophen-2-yl)acrylonitrile* (**PT**). Green solid, Yield: >98%, m.p. 118–119 °C, FTIR (ATR, cm^−1^), 3108 (C–H), 2208 (CN), 1590 (C=C), 1489 (C–S). ^1^H-NMR (CDCl_3_, 500 MHz) δ 7.6–7.7 (2H, m, H8 and H11), 7.4–7.6 (5H, m, H1, H3, H4, H6 and H13), 7.1–7.2, (1H, dd *J* = 3.78, 5.05 Hz, (H12). ^13^C-NMR (CDCl_3_,125 MHz) δ 132.3, 123.2, 127.2, 132.9, 107.2, 134.6, 137.8, 130.6, 128.1, 132.9, 117.9 (CN). HRMS–TOF (ESI, *m*/*z*): [M + H]^+^ Found: 289.9634 for C_13_H_8_BrNS; [M + H]^+^ Calcd.: 289.9640.

*(E)-3-(5-Bromothiophen-2-yl)-2-(thiophen-2-yl)acrylonitrile* (**TT**). Yellow solid, Yield: >98%, m.p. 116–118 °C. FTIR (ATR, cm^−1^), 3100 (C–H), 2214 (CN), 1585 (C=C), 1433 (C–S). ^1^H-NMR (CDCl_3_, 500 MHz): δ 7.24–7.34 (4H, m, H3, H8, H12 and H10), 7.0–7.1 (2H, m, H7 and H11), ^13^C-NMR (CDCl_3_, 125 MHz) δ 138.4, 103.4, 132.7, 139.2, 118.2, 128.34, 131.2, 127.4, 126.4, 130.7, 116.9 (CN). HRMS–TOF (ESI, *m*/*z*): [M + H]^+^ Found: 295.9197 for C_11_H_6_BrNS_2_; [M + H]^+^ Calcd.: 295.9204.

#### 3.3.2. General Method for Suzuki Reaction

In a flask of 20 mL are placed the diboronic acid and the corresponding aryl halides; subsequently, 3 mol equivalents of PIVOK is added as well as 0.05% mol of the catalyst tetrakistriphenylphosphine palladium Pd(PPh_3_)_4_. The mixture was placed under nitrogen, then 5 mL of dimethylacetamide (DMA) is added, the mixture was stirred for 2 min and then heated up in a microwave oven at 110 °C with a power of 100 W, after 1 h the reaction was cooled down to 50 °C and 50 mL of cold EtOH are added, a solid precipitate is observed, which is filtered and washed first with 50 mL of EtOH and then with 50 mL of hexane. The solid was recovered and recrystallized using a mixture of ketone/EtOH 40:60.

*1,3-bis(4-(Biphenylamino)phenyl)-5-(2-ethylhexyl)-4H-thieno[3,4-c]pyrrole-4,6(5H)-dione* (**TPA-TPD**). Orange solid, Yield: 70%, m.p. 178–179 °C. FTIR (ATR, cm^−1^), 3068 (C-H), 1687 (C=O), 1494 (C–S). ^1^H-NMR (CDCl_3_, 500 MHz) δ 7.95–8.05 (4H, m), 7.27–7.40 (8H, m), 7.00–7.23 (16H, m), 3.54 (2H, d, *J* = 7.2 Hz), 1.75–1.91 (1H, m), 1.25–1.40 (8H, m), 0.82–0.98 (6H, m); ^13^C-NMR (CDCl_3_, 125 MHz) δ 163.4, 149.3, 146.8, 144.1, 129.4, 129.0, 128.6, 125.4, 124.0, 123.7, 121.4, 42.4, 38.2, 30.6, 28.6, 23.9, 23.0, 14.1, 10.5. HRMS–TOF (ESI, *m*/*z*): [M + H]^+^ Found: 752.3307 for C_50_H_45_N_3_O_2_S; [M + H]^+^ Calcd.: 752.3311. 

*(Z)-2-(4′-(Diphenylamino)-[1,1′-biphenyl]-4-yl)-3-(thiophen-2-yl)acrylonitrile* (**TPA-PT**). Orange solid, Yield: 84%, m.p. 174–175 °C, FTIR (ATR, cm^−1^), 2928 (C–H), 2207 (CN), 1586 (C=C), 1485 (C–S). ^1^H-NMR (CDC_l3_, 500 MHz) δ 7.6–7.7 (6H, m, H10, H10′, H11, H11′, H14 and H16), 7.50 (2H, d, *J* = 8.6 Hz, H7 and H7′), 7.5 (1H, d, *J* = 5.0 Hz, H18), 7.23–7.33 (5H, m, H2, H2′, H17, H22 and H22′), 7.10–7.20 (6H, m, H3, H3′, H6, H6′, H22, and H22′), 7.01–7.09 (2H, t, *J* = 6.9 Hz, H1 and H23); ^13^C-NMR (CDCl_3_, 125 MHz,) δ 147.8, 147.6, 141.3, 138.2, 133.5, 132.3, 132.3, 130.0, 129.4, 127.9, 127.7, 127.2, 126.2, 124.7, 124.7, 123.6, 123.3, 118.2, 108.1. HRMS–TOF (ESI, *m*/*z*): [M + H]^+^ Found: 455.1573 for C_31_H_22_N_2_S; [M + H]^+^ Calcd.: 455.1565. 

*(E)-3-(5-(4-(Diphenylamino)phenyl)thiophen-2-yl)-2-(thiophen-2-yl)acrylonitrile* (**TPA-TT**). Brown solid, Yield: 78%, m.p. 157–158 °C. FTIR (ATR, cm^−1^), 3100 (C–H), 2211 (CN), 1587 (C=C), 1433 (C–S). ^1^H-NMR (CDCl_3_, 500 MHz) δ 7.48–7.53 (3H, m, H10, H11 and H18), 7.42 (1H, s, H13), 7.18–7.33 (7H, m, H2, H2′, H6, H6′, H16, H22 and H22′), 7.23–7.33 (5H, m, H2, H2′, H17, H22 and H22′), 7.03–7.09 (9H, m, H1, H3, H3′, H7, H7′, H17, H21, H21′ and H23); ^13^C-NMR (CDCl_3_, 125 MHz) δ 149.9, 148.3, 147.0, 139.0, 135.0, 134.0, 132.1, 129.9, 129.3, 128.1, 126.7, 126.4, 125.5, 124.8, 123.4, 122.8, 122.6, 117.2, 101.5. HRMS–TOF (ESI, *m*/*z*): [M + H]^+^ Found: 461.1125 for C_19_H_20_N_2_S_2_; [M + H]^+^ Calcd.: 461.1146. 

#### 3.3.3. Direct Arylation

In a flask of 20 mL are placed 0.1 mmol of 1,3-dibromo-5-(2-ethylhexyl)-4*H*-thieno[3,4-*c*]pyrrole-4,6(5*H*)-dione (TPD) and then 0.2 mmol of TPA-PT or TPA-TT are added; subsequently 3 molar equivalents of PIVOK are added as well as 0.05 mol % of catalyst palladium (II) acetate Pd(OAc)_2_, the mixture was placed under nitrogen atmosphere, then 5 mL of DMA are added. The mixture was stirred for 2 min and then heated up in a microwave oven at 110 °C with a power of 100 W. After 1 h, the reaction was cooled down to 50 °C and 50 mL of cold EtOH was added and a solid precipitate is observed, which is filtered and washed first with 50 mL of EtOH and then with 50 mL of hexane. The solid was recovered and recrystallized using a mixture of acetone/EtOH 40:60.

*(2Z,2′Z)-3,3′-(5,5′-(5-(2-Ethylpentyl)-4,6-dioxo-5,6-dihydro-4H-thieno[3,4-c]pyrrole-1,3-diyl)bis(thiophene-5,2-diyl))bis(2-(4′-(diphenylamino)-[1,1′-biphenyl]-4-yl)acrylonitrile)* (**TPA-PT-TPD**). Dark solid, Yield: 60%, m.p. 173–175 °C. FTIR (ATR, cm^−1^), 3010 (C–H), 2219 (CN), 1696 (C=O), 1588 (C=C), 1432 (C–S). ^1^H-NMR (C_6_D_6_, 500 MHz) δ 7.7–7.81 (12H, m), 7.51–7.62(4H, m), 7.23–7.33 (10H, m), 7.10–7.20 (12H, m), 7.01–7.09 (4H, m), 3.57 (2H, s broad), 1.81–1.93 (1H, m), 1.23–1.47 (8H, m), 0.81-1.02 (6H, m). ^13^C-NMR (CDCl_3_, 125 MHz) δ 159.5, 145.2, 144.8, 138.6, 133.2, 132.7, 130.2, 130.5, 129.9, 128.7, 128.2, 127.6, 126.9, 124.2, 123.4, 122.3, 120.5, 120.8, 106.7, 106.5, 40.3, 35.7, 28.2, 26.2, 21.4, 20.7, 11.7, 8.0, 159.7, 115.0 (CN). HRMS–TOF (ESI, *m*/*z*): [M + H]^+^ Found: 1169.7796 for C_76_H_59_N_5_O_2_S_3_; [M + H]^+^ Calcd.: 1169.7783. 

*(2E,2′E)-2,2′-(5,5′-(5-(2-Ethylpentyl)-4,6-dioxo-5,6-dihydro-4H-thieno[3,4-c]pyrrole-1,3-diyl)bis(thiophene-5,2-diyl))bis(3-(5-(4-(diphenylamino)phenyl)thiophen-2-yl)acrylonitrile)* (**TPA-TT-TPD**). Dark solid, Yield: 70%, m.p. 179–182 °C. FTIR (ATR, cm^−1^), 2926 (C–H), 2211 (CN), 1699 (C=O), 1587 (C=C), 1493 (C–S). ^1^H-NMR (C_6_D_6_, 500 MHz) δ 7.68 (1H, s broad), 7.16–7.60 (20H, m), 6.79–7.16 (17H, m), 3.57 (2H, s broad), 1.81–1.93 (1H, m), 1.23–1.47 (8H, m), 0.81–1.02 (6H, m). ^13^C-NMR (CDCl_3_, 125 MHz) δ 150, 148.4, 147.1, 147.0, 142.8, 135.5, 135.4, 132.7, 132.0, 130.5, 129.5, 128.9, 126.9, 126.2, 125.1, 123.8, 122.6, 122.5, 122.3, 116.5, 42.9, 38.3, 30.7, 28.7, 23.9, 23.3, 14.3, 10.6, 162.4 (C=O), 100 (CN). [M + H]^+^ Found: 1128.4051 for C_72_H_55_N_5_O_2_S_5_; [M + H]^+^ Calcd.: 1181.2959.

## 4. Conclusions

**TPA-TPD**, **TPA-PT-TPD** and **TPA-TT-TPD** molecules were synthesized by using microwave assistance and thus decreasing the reaction time from 48 to 1 h. It could help to reduce costs in the synthesis of semiconductor materials. These small molecules were used in solution processed OSCs fabrication for the first time; the largest V_OC_ was almost of 1 V for those cells based on **TPA-TPD**:PC71BM; while J_SC_ value was 7.9 mA/cm^2^ and thus PCE = 2.6%. Through AFM measurements, it was observed that one of the factors involved in the low photovoltaic parameters of OSCs based on **TPA-PT-TPD**:PC71BM and **TPA-TT-TPD**:PC71BM is an irregular morphology of the formed films. They showed a roughness of 22 and 28 nm, respectively (for films based on **TPA-TPD**, roughness was just 10 nm). Although power energy efficiencies of our OSCs based on the described SMs containing TPD are still lower than those for OSCs based on other SMs containing this same fragment, our SMs synthesis procedure is easier and faster than in other reports. Furthermore, our OSCs were fabricated and tested under regular atmosphere and by using as cathode the eutectic alloy Field’s metal, which is easily free vacuum deposited. However, it is necessary to improve film morphology; probably it could be through evaporation or by improving solubility of the molecules by adding alkyl chains.

## Figures and Tables

**Table 1:** Optical and Electrochemical properties of **TPA-TPD**, **TPA-PT-TPD** and **TPA-TT-TPD**.

SM	λ_max_ (nm) ^a^	λ_max_ (nm) ^b^	ε (M^−1^ cm^−1^)	E_g_^opt^ (eV) ^c^	E_H_ (eV)	E_L_ (eV)	E_g_^elec^ (eV)
**TPA-TPD**	433	434	52,382	2.48	−5.34	−2.87	2.47
**TPA-PT-TPD**	495	500	51,200	1.90	−5.24	−3.05	2.19
**TPA-TT-TPD**	527	551	52,892	1.74	−5.17	−3.19	1.98

^a^ Measured absorption in chloroform solution at room temperature. ^b^ Thin solid film (~100 nm) spin-coated from chloroform solution onto glass slides. ^c^ Calculated from the absorption band edge of thin films, E_g_^opt^ = 1240/λ_edge_ (eV).

**Table 2:** Photovoltaic performance of OSCs devices based on **TPA-TPD**, **TPA-PT-TPD** and **TPA-TT-TPD**, under the ITO/PEDOT:PSS/SM:PC71BM/PFN/FM structure.

SM	Ratio D:A	Thickness ^(a)^ (nm)	J_SC_ (mA/cm^2^)	V_OC_ (V)	FF	PCE %
**TPA-TPD**	1:4	~78	7.3 ± 0.5 (7.9)	0.97 ± 0.02 (0.99)	0.33 ± 0.01 (0.34)	2.32 ±0.28 (2.6)
**TPA-PT-TPD**	1:3	~80	3.1 ± 0.4 (3.5)	0.80 ± 0.02 (0.82)	0.27 ± 0.01 (0.28)	0.67 ± 0.12 (0.79)
**TPA-TT-TPD**	1:2	~79	3.6 ± 0.41 (4.1)	0.74 ± 0.14 (0.89)	0.30 ± 0.03 (0.33)	0.81 ± 0.10 (0.91)

^(a)^ Active film. The average of PCE was determined from eight cells manufactured under the same conditions. Numbers in parenthesis represent the best value of 8 tested devices.

**Table 3:** Comparison of photovoltaic parameters for OSCs based on **iI(TPA)_2_** and **TPA-DPP** with those measured in this work based on **TPA-TPD**.

Donor	Acceptor	D:A (*w*/*w*)	J_SC_ (mA/cm^2^)	V_OC_ (V)	FF	PCE (%)	HOMO/LUMO (eV)	Ref.
**iI(TPA)_2_**	PC61BM	1:3	2.9	0.78	0.37	0.84	−5.30/−3.61	[33]
**TPA-DPP**	PC71BM	1:4	4.3	0.73	0.30	1.0	−5.07/−3.44	[34]
**TPA-TPD**	PC71BM	1:4	7.9	0.97	0.34	2.6	−5.34/−2.86	This work

## Supplementary Materials

Supplementary materials are available online.
