# Supplementary material for: Small Molecules Derived from Thieno[3,4-c]pyrrole-4,6-dione (TPD) and Their Use in Solution Processed Organic Solar Cells
Source: Molecules. 2017 Sep 30;22(10):1607. doi: 10.3390/molecules22101607 (PMC6151745; doi:10.3390/molecules22101607)
Supplement: Supplementary file 1 [file molecules-22-01607-s001.pdf]

Supporting Information

Small Molecules Derived From Thieno[3,4-*c*]Pyrrole-4,6-Dione (TPD) and Their Use in Solution Processed Organic Solar Cells

Cesar Garcias-Morales [1, *,†], Daniel Romero-Borja [1, ¥], José-Luis Maldonado [1,*], Arián E. Roa [1, §], Mario Rodríguez [1], J. Pablo García-Merinos [2] and Armando Ariza-Castolo [3]

[1] Research Group of Optical Properties of Materials (GPOM), Centro de Investigaciones en Óptica, A.P. 1-948, 37000 León, Gto., México; cgarcias@uadec.edu.mx (C. G-M); ad.romero.borja@cio.mx (D R-B); jlmr@cio.mx (J-L M); espinosa.arian@gmail.com (A E. R); mrodri@cio.mx (M. R).

[2] Instituto de Investigaciones Químico Biológicas Universidad Michoacana de San Nicolás de Hidalgo Edificio B-1; Ciudad Universitaria, Morelia, Michoacán C.P. 58030, México; jpgarciam@gmail.com (J. P. G-M).

[3] Departamento de Química, Centro de Investigación y de Estudios Avanzados del Instituto Politécnico Nacional, Avenida Instituto Politécnico Nacional 2508 Colonia San Pedro Zacatenco, C.P. 07360, México, D.F., México; aariza@cinvestav.mx (A. A-C).

[*]**E-mail:** cgarcias@uadec.edu.mx, jlmr@cio.mx

Present address: [†] Departamento de Química Orgánica, Facultad de Ciencias Químicas, Universidad Autónoma de Coahuila, Ing. José Cárdenas Valdez, República, 25280 Saltillo, Coah. México.

[¥] Centro de Investigación en Química Aplicada, Boulevard E. Reyna 140, 25100 Saltillo, México.
[§] Instituto de Investigaciones en Materiales, Universidad Nacional Autónoma de México, Del. Coyoacán, C.P. 04510 Ciudad de México, México.

1. Measurements

Fourier transform infrared (FTIR) spectra were acquired using Varian 640 spectrophotometer through the ATR method. The absorption spectra were measured using a PerkinElmer/Lambda 900 model UV/VIS/NIR spectrophotometer, for solution spectra the SMs were dissolved in chloroform, while in solid state SMs were supported on glass substrates by spin-coating. ^{1}H and ^{13}C NMR spectra were recorded at 21 ± 1 °C using a JEOL ECA 500 MHz spectrometer equipped with a 5 mm multinuclear pulse-field gradient probe. Spectra were recorded in $CDCl_3$ solution (0.9 mmol of the compound per 0.5 mL of solvent). The chemical shifts were referenced to tetramethylsilane $(CH_3)_4Si$, which served as an internal standard ($\delta^{1}H = 0$, $\delta^{13}C = 0$).

2. Details of the calculations and computational methods

The computational chemistry calculations were performed using the Gaussian 09 package, and molecular visualization was performed with ChemCraft 1.7 (2013) software. The functional and basis set used were selected based on the results published by Dixon D. A. et al., [1]. In this work, authors concluded that for the calculation of molecular orbitals energies and electronic density distribution of organic molecules, the use of the exchange correlation BL3YP functional and the 6-31 + G * basis set is enough to calculate the Kohn-Sham (HOMO and LUMO) orbitals and to correlate them with the molecular properties.

To have an idea of the electronic transitions, which are due the experimental UV-vis bands, TD-DFT was carried out. In the Table S1 are listed the excited-state energies, the corresponding oscillator strength (f) and the configuration of the transition, calculated by TD-DFT. Analyses showed that for three molecules, the experimental red-shifted absorption bands are provoked for HOMO→LUMO (H→L) transitions. These electronic transitions correspond to symmetric intramolecular charge transfer from donor to acceptor groups contained in the molecular structure.

Table S1 Excited-state vertical transition energies (Evert, eV), oscillator strengths (arbitrary units), and electronic configuration of the first excited state, as determined with TD-DFT at the B3LYP/6-31G(d) level of theory

Compound	*λabs (nm)*	*Eex (eV)*	*f*	*Configuration*
TPA-TPD	*468.72*	*2.6452*	*1.1950*	*H→L (98.58%)*
	437.66	*2.8329*	*0.1152*	*H →L +1(96.87%)*
	403.79	*3.0705*	*0.1117*	*H-1→L (96.01%)*
TPA-TT-TPD	*644.70*	*1.9231*	*3.0177*	*H→L (95.89%)*
	567.29	*2.1855*	*0.0332*	*H-1→L (83.04%)*
	543.63	*2.2807*	*0.0332*	*H →L +1(82.99%)*
TPA-PT-TPD	*668.93*	*1.8535*	*1.3737*	*H→L (96.48%)*
	639.42	*1.9390*	*0.0095*	*H-1→L (99.01%)*
	547.71	*2.2637*	*0.0010*	*H →L +1(98.30%)*

3. Details for Cyclic voltammetry

Cyclic voltammetry (CV) was performed using an advanced electrochemical system, PARSTAT 2273 potentiostat; a platinum electrode, a silver wire, and 0.1 M tetrabutylammonium perchlorate (TBAP) were used as the working electrode, counter electrode, and supporting electrolyte, respectively. $Ag^+/AgNO_3$ was used as the reference electrode (at room temperature) and calibrated against a standard hydrogen electrode (SHE) using ferrocene/ferrocenium (Fc/Fc^+). Silver was used in this experiment as a known reference to calculate the E_{ox} or E_{red} values. The energy levels estimations can be done with the empirical relation $E_{LUMO} = -[(E_{red} + 4.4) + 0.22]$ eV or $E_{HOMO} = -[(E_{ox} + 4.4) + 0.22]$ eV. CV measurements were carried out in $TBAP/CH_2Cl_2$ solution under a nitrogen atmosphere at a scan rate of 50 mVs^{-1}.

4. Photovoltaic device fabrication and characterization

Mixed solutions for the active film were prepared by dissolving 35 mg/mL of TPA-TPD:$PC_{71}BM$, TPA-PT-TPD:$PC_{71}BM$ and TPA-TT-TPD:$PC_{71}BM$ in chlorobenzene with blend ratios of 1:4, 1:3 and 1:2 (w/w D:A), respectively. The bulk heterojunction photovoltaic devices were fabricated using a common process at ambient conditions under the ITO/PEDOT:PSS/SMs:$PC_{71}BM$/PFN/FM architecture [2−6]. Prior to device

fabrication, the indium tin oxide (ITO) coated glass substrates were sequentially cleaned in detergent, de-ionized water, acetone and isopropanol. The hole-injection buffer layer of PEDOT:PSS, was spin-coated on the ITO-coated glass substrate at 5000 RPM for 60 s (~ 40 nm of thickness), then, this film was thermally treated for 20 min at 120 °C in a hot plate. Each SM:$PC_{71}BM$ solution was spin-coated onto PEDOT:PSS-coated substrates (~100 nm of thickness). Then, these films were subjected to thermal annealing for 20 min at 80 °C in a hot plate; the PFN interlayer material was dissolved in methanol (concentration: 2 mg/mL) under the presence of a small amount of acetic acid and spin-coated on top of the active layer (film thickness ~ 8 nm). Finally, Field's metal was deposited by drop-wise on the patterned substrate. Current density–voltage (J–V) of the photovoltaic devices were measured using a Keithley 2450 source measure unit and a solar simulator Sciencetech calibrated at 100 $mWcm^{-2}$ (AM 1.5 conditions).

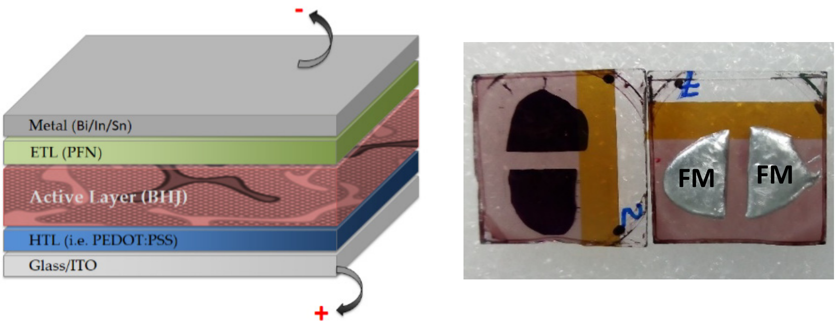

Figure S1. Architecture of OSCs, ITO/PEDOT:PSS/SMs:$PC_{71}BM$/PFN/FM.

5. Synthesis

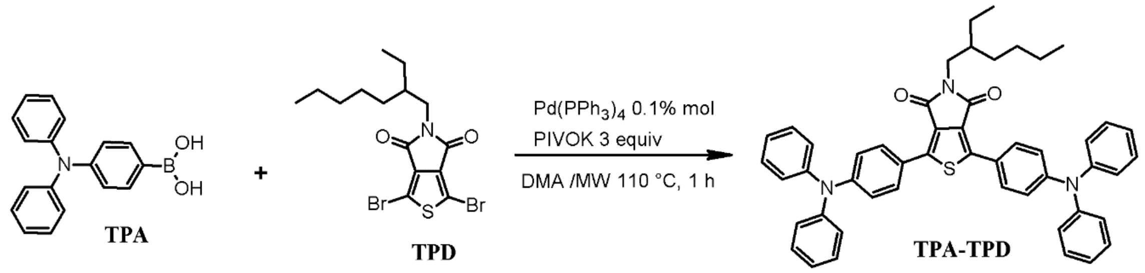

Scheme S1. Synthetic route for **TPA-TPD**

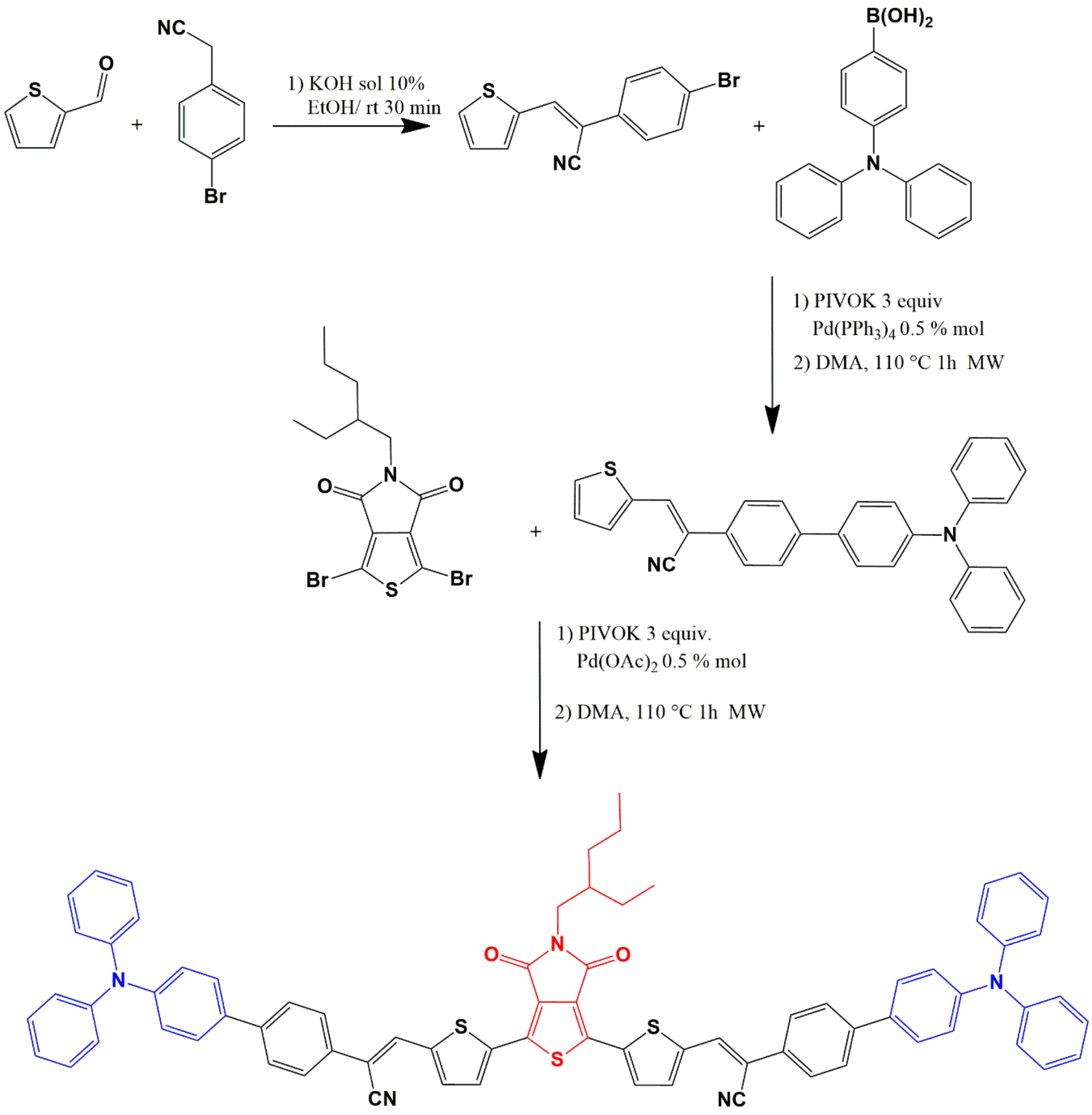

Scheme S2. Synthetic route for **TPA-PT-TPD**

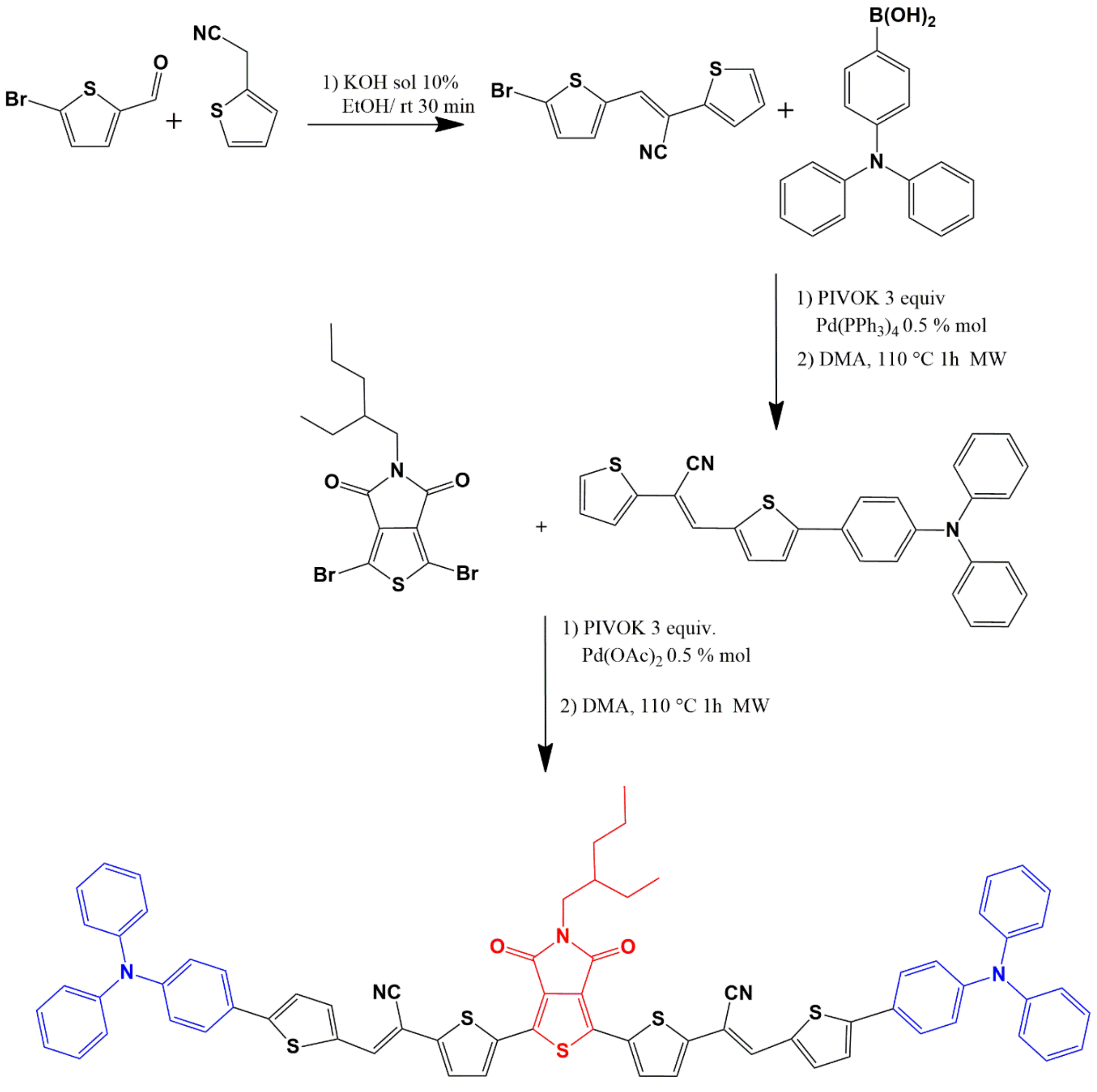

Scheme S3. Synthetic route for **TPA-TT-TPD**

6. Planarity Analysis of SMs by DFT

Table *S1* shows the torsional angles for **TPA-TPD**, **TPA-PT-TPD** and **TPA-TT-TPD** obtained from the geometry optimization by DFT. Values of φ_1 and φ_1' close to zero (0.5−1.5 °) indicate that the portion of the molecule, which is attached directly to TPD fragment is coplanar, it is due to the C−H···O or S···O intermolecular interaction (Figures S2−S5). [7] This molecular planarity, favors the electron delocalization of the molecules and therefore the energy of internal charge transfer of donor to the acceptor is lower. For **TPA-PT-TPD** geometry is not completely coplanar because the phenyl TPA is outside of plane (φ_2 and φ_2', 33.0−33.5 °), it increases the energy barrier for electron delocalization, so the electron density on HOMO is localized in the donor fragment, while the electron density LUMO is confined in the acceptor fragment.

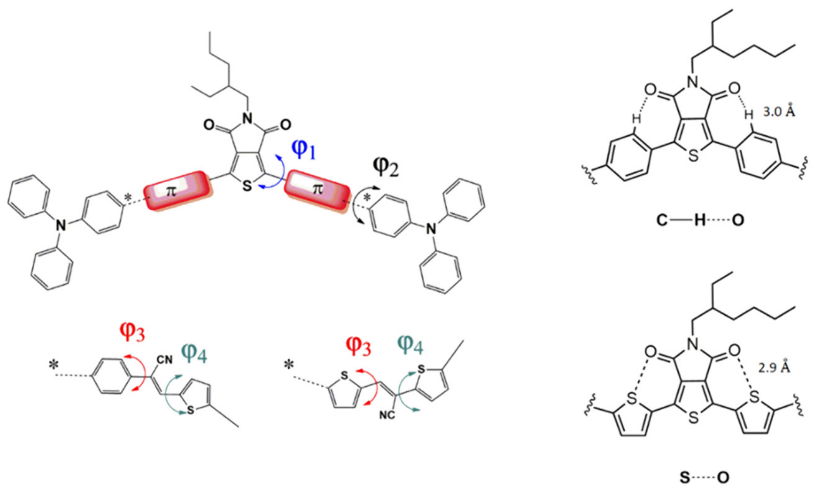

Figure S2. Torsional angles (φ) for **TPA-TPD**, **TPA-PT-TPD** and **TPA-TT-TPD** from theoretical simulations using DFT at the B3LYP/6-31G(d) level.

Table S1. Dihedral angles (in degrees) of SM**s** from theoretical simulations by using DFT at the B3LYP/6-31G(d) level.

SM	φ_1	φ_2	φ_3	φ_4	φ_1'	φ_2'	φ_3'	φ_4'
**TPA-TPD**	0.6				0.5			
**TPA-PT-TPD**	0.7	33.0	1.5	21.0	0.7	33.5	1.6	21.5
**TPA-TT-TPD**	1.5	20.2	1.3	7.3	1.1	20.3	1.5	7.4

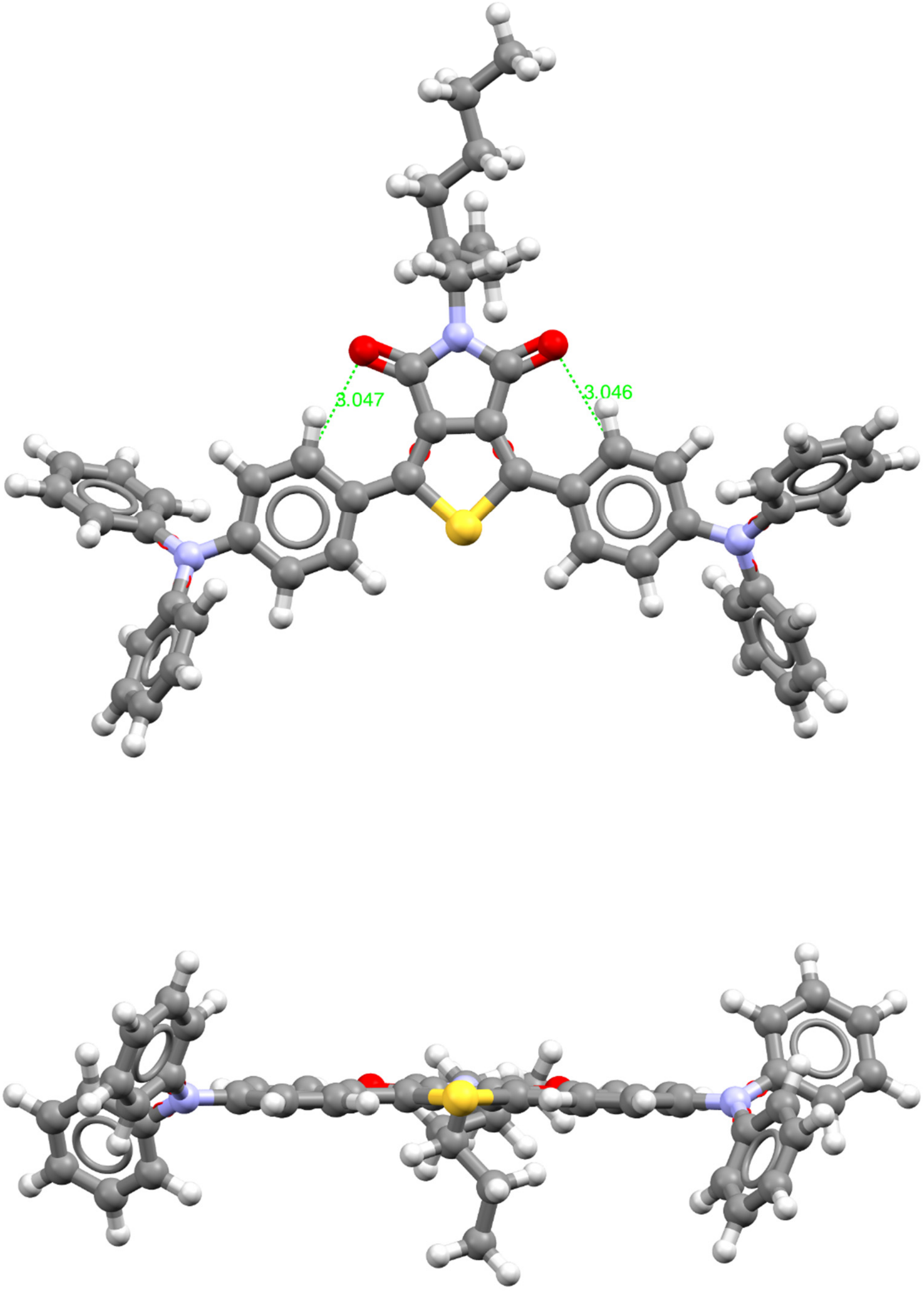

Figure S3. The optimized geometries of **TPA-TPD** using DFT at the B3LYP/6-31G(d) level.

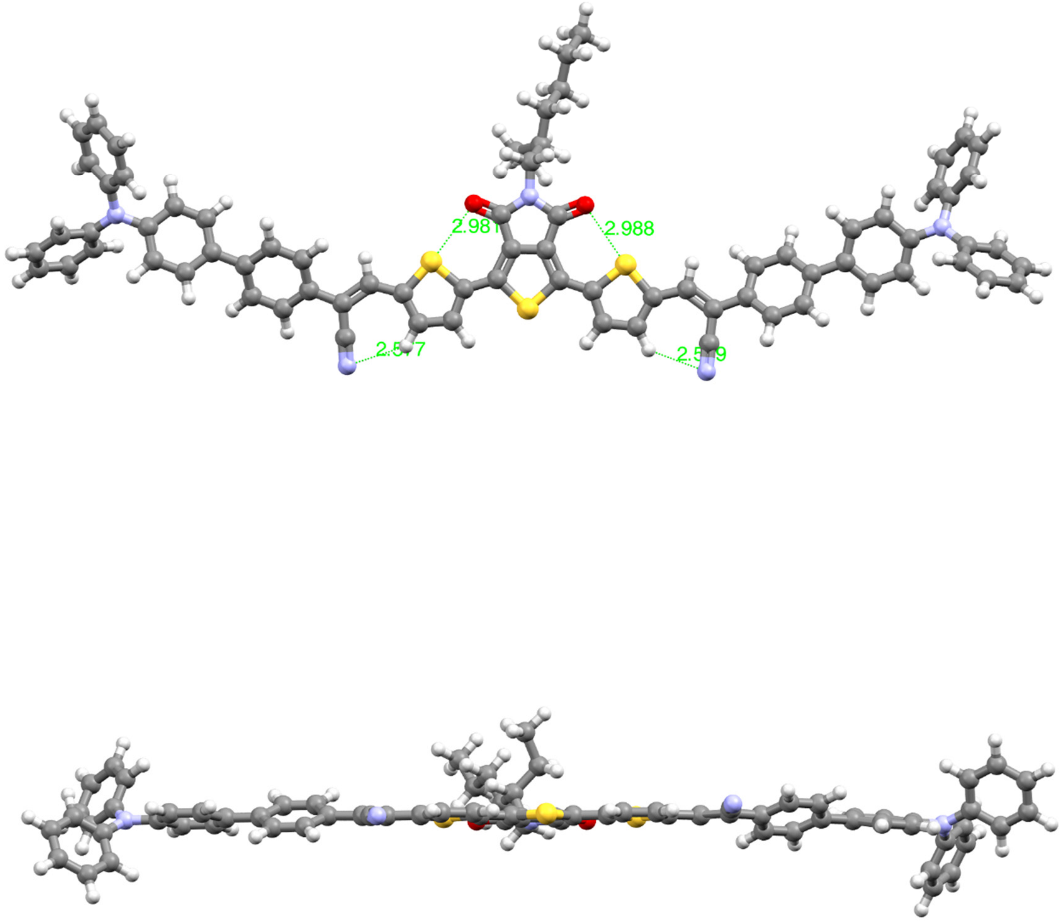

Figure S4. The optimized geometries of **TPA-PT-TPD** using DFT at the B3LYP/6-31G(d) level.

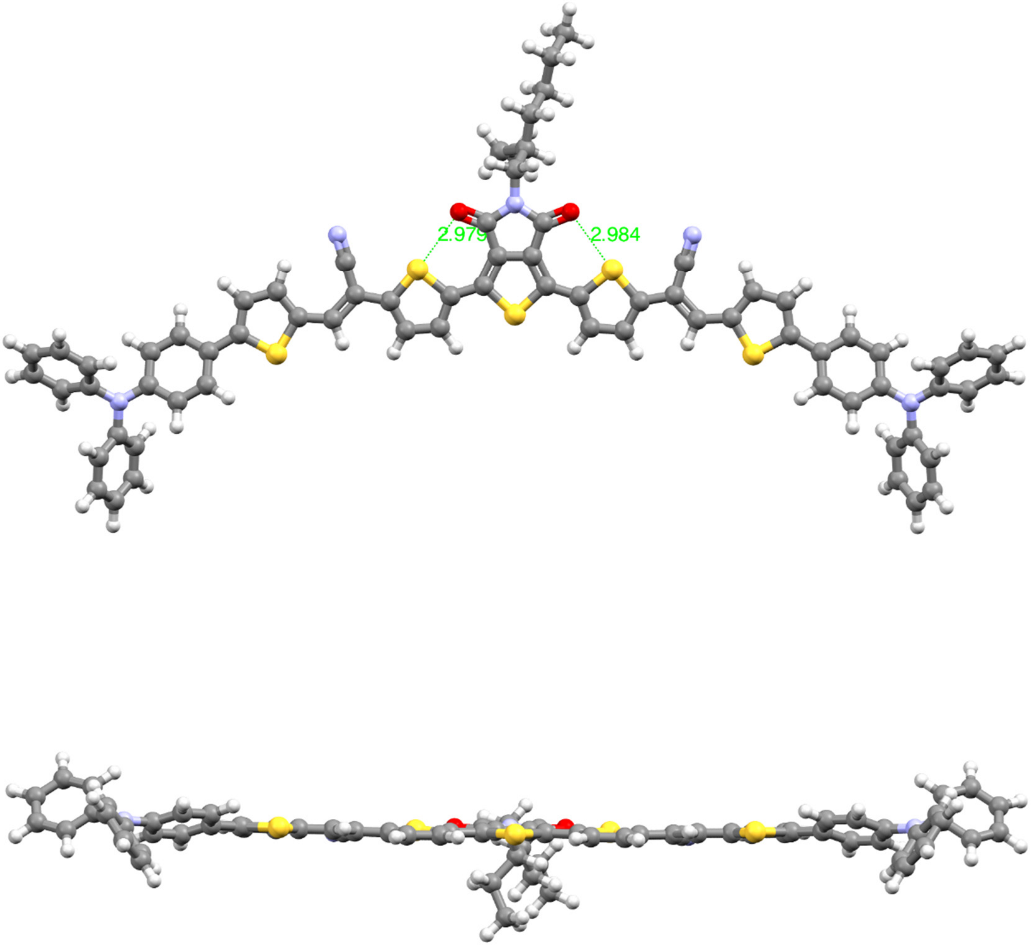

Figure S5. The optimized geometries of **TPA-TT-TPD** using DFT at the B3LYP/6-31G(d) level.

Table S2. Comparison of the photovoltaic properties for OSCs based on TPA-TPD:$PC_{71}BM$ with different donor-acceptor weight ratios, measured under an illumination of AM 1.5 G, 100 mW cm^{-2}.

ID Device	donor-acceptor w/w	J_{sc} (mA/cm^2)	V_{oc} (V)	FF (%)	PCE (%)
C1	1:1	-3.2	0.76	31	0.74
C2		-2.7	0.66	32	0.58
C3	1:2	-4.1	0.79	30	0.97
C4		-3.2	0.82	31	0.83
C5	1:3	-4.7	0.65	32	0.97
C6		-4.6	0.72	31	1.05
C7	1:4	-5.0	0.82	31	1.25
C8		-5.0	0.77	29	1.13

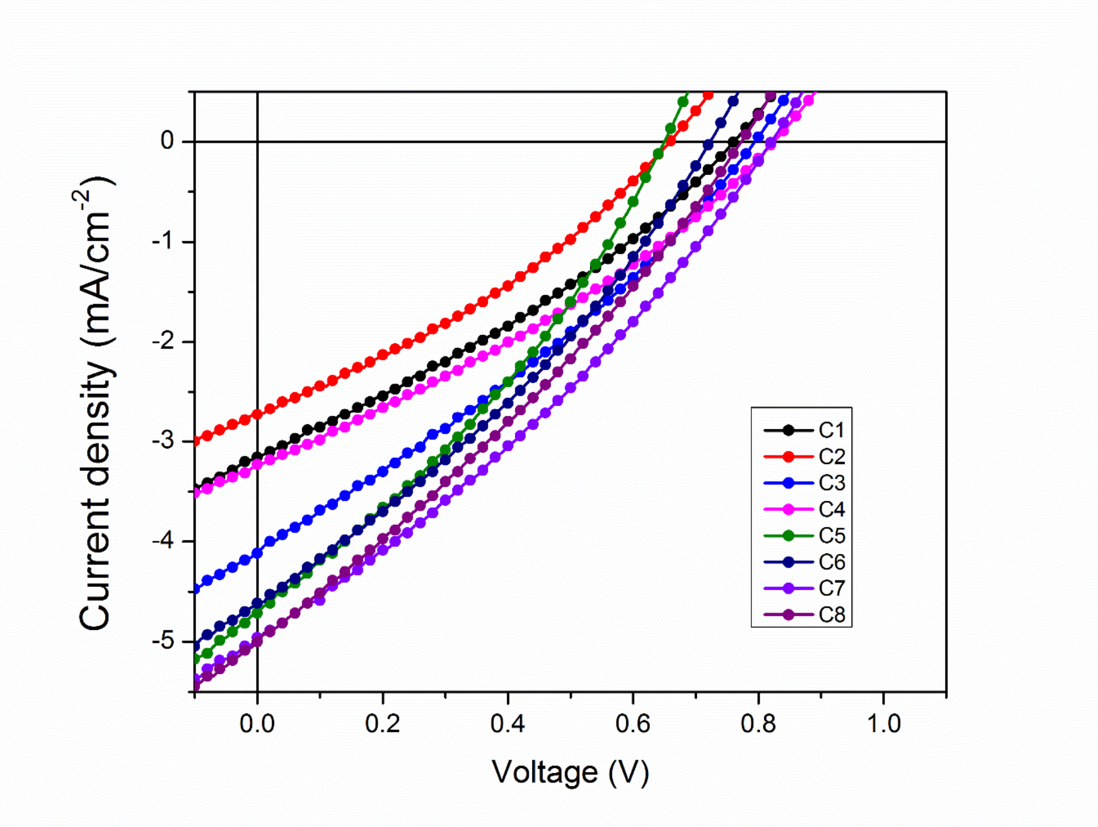

Figure S6. *J-V* curve of the OSCs based on TPA-TPD:$PC_{71}BM$ with different donor-acceptor weight ratios.

Table S3. Comparison of the photovoltaic properties of the OSCs based on TPA-TT-TPD:$PC_{71}BM$ with different donor-acceptor weight ratios, measured under an illumination of AM 1.5 G, 100 mW cm^{-2}.

ID	D/A Ratio	J_{sc} (mA/cm^2)	V_{oc} (V)	FF (%)	PCE (%)
C1	1:1 w/w	-2.0	0.74	28	0.41
C2		-2.3	0.71	29	0.46
C3	1:2 w/w	-3.9	0.79	29	0.91
C4		-3.6	0.66	31	0.75
C5	1:3 w/w	-2.9	0.29	27	0.23
C6		-2.8	0.34	31	0.29

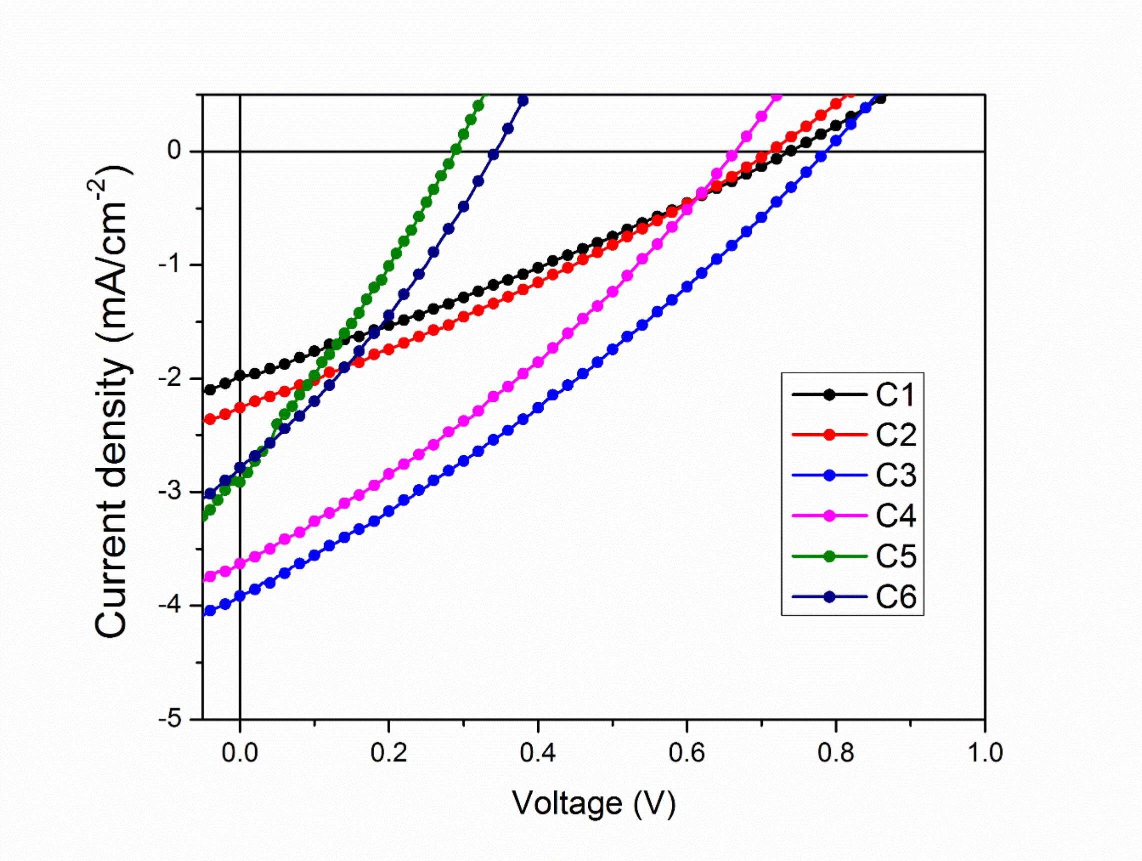

Figure S7. *J-V* curve of the OSCs based on TPA-TT-TPD:$PC_{71}BM$ with different donor-acceptor weight ratios.

Table S4. Average of photovoltaic properties of the OSCs based on TPA-TPD:$PC_{71}BM$ with 1:4 w/w D:A ratios, measured under an illumination of AM 1.5 G, 100 mW cm^{-2}.

ID	Jsc (mA/cm^2)	Voc (V)	FF (%)	PCE (%)
C01	-7.7	0.97	33	2.45
C02	-6.7	0.99	34	2.30
C03	-7.8	0.97	33	2.51
C04	-7.4	0.95	32	2.24
C05	-6.4	0.99	34	2.17
C06	-7.9	0.97	34	2.57
C07	-7.0	0.96	33	2.20
C08	-7.1	0.94	32	2.11
Average	-7.25	0.97	33.13	2.32

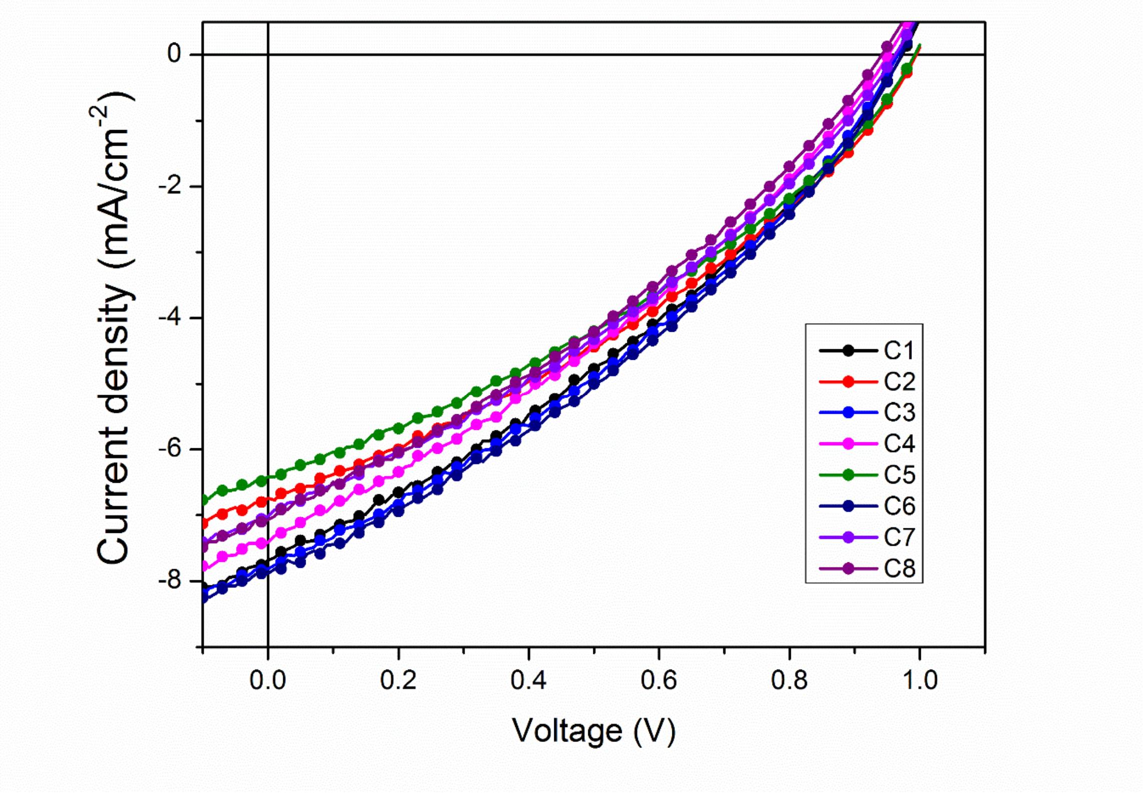

Figure S8. *J-V* curve of the OSCs based on TPA-TPD:$PC_{71}BM$ 1:4 w/w D:A, average from 8 cells

Table S5. Average of photovoltaic properties of the OSCs based on TPA-PT-TPD:$PC_{71}BM$ with 1:3 w/w D:A ratios, measured under an illumination of AM 1.5 G, 100 mW cm^{-2}.

ID Device	J_{sc} (mA cm^{-2})	V_{oc} (V)	FF (%)	PCE (%)
C01	-3.4	0.82	28	0.77
C02	-2.7	0.80	27	0.59
C03	-3.1	0.80	27	0.67
C04	-3.1	0.80	28	0.69
C05	-3.0	0.79	26	0.63
C06	-3.5	0.82	28	0.79
C07	-3.1	0.79	27	0.65
C08	-2.9	0.78	26	0.60
Average	-3.1	0.80	27.13	0.67

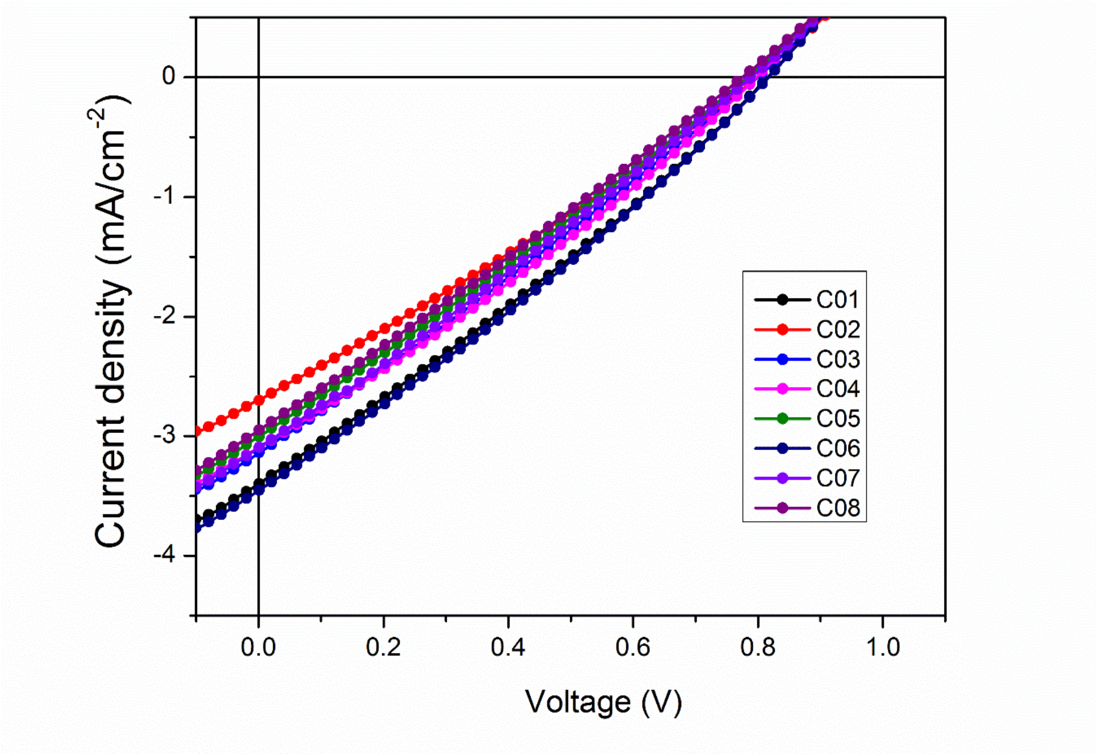

Figure S9. *J-V* curve of the OSCs based on TPA-PT-TPD:$PC_{71}BM$ 1:3 w/w D:A, average from 8 cells

Table S6. Average of photovoltaic properties of the OSCs based on TPA-TT-TPD:$PC_{71}BM$ with 1:2 w/w D:A ratios, measured under an illumination of AM 1.5 G, 100 mW cm^{-2}.

ID	J_{sc} (mA/cm^2)	V_{oc} (V)	FF (%)	PCE (%)
C1	-3.7	0.71	33	0.87
C2	-4.1	0.77	28	0.91
C3	-3.4	0.68	33	0.78
C4	-3.9	0.79	29	0.91
C5	-3.6	0.66	31	0.75
C6	-3.2	0.82	31	0.83
C7	-2.9	0.89	30	0.77
C8	-3.7	0.65	28	0.69
Average	-3.56	0.75	30.38	0.81

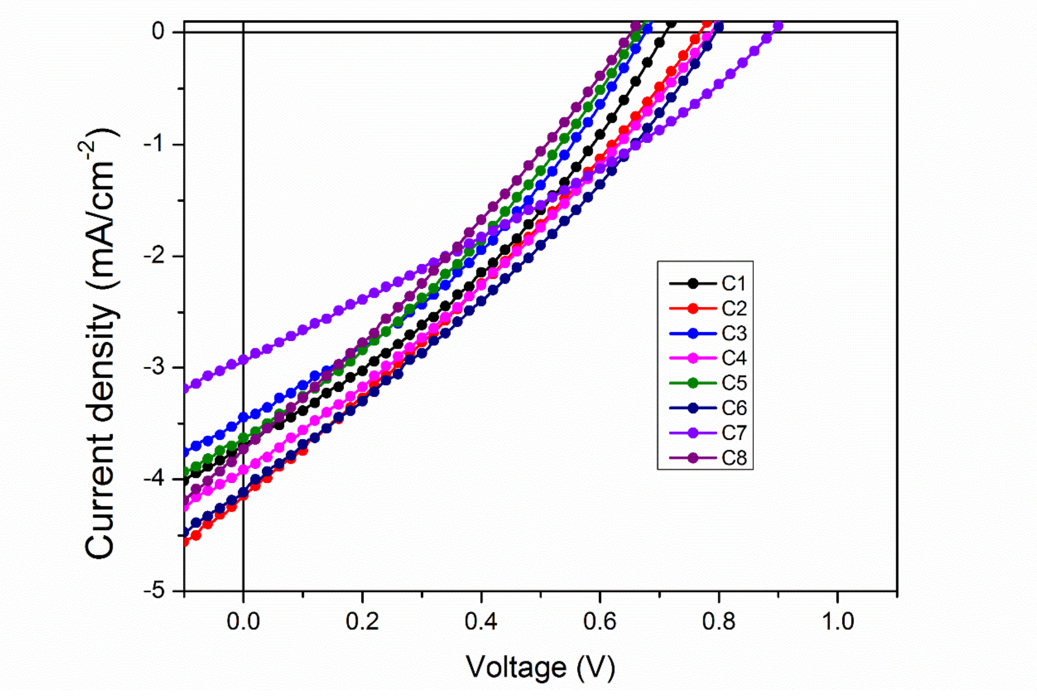

Figure S10. *J-V* curve of the OSCs based on TPA-TT-TPD:$PC_{71}BM$ 1:2 w/w D:A, average from 8 cells

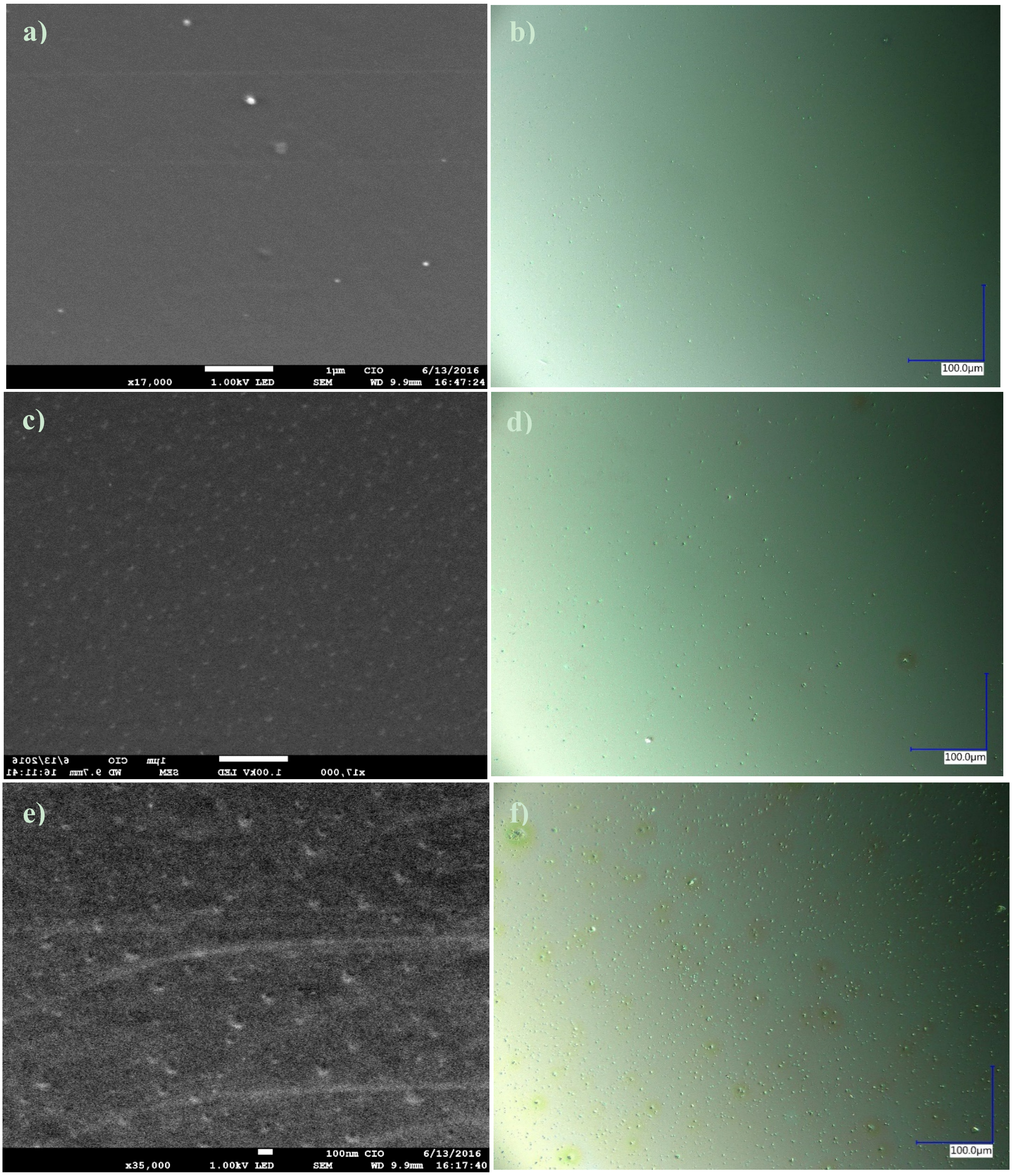

Figure S11. a), c), and e) correspond to SEM images of the active layers SM:$PC_{71}BM$ of **TPA-TPA, TPA-PT-TPD** and **TPA-TT-TPD** respectively. b), d), and f) correspond to optical microscopy images of the active layers SM:$PC_{71}BM$ of **TPA-TPA**, **TPA-PT-TPD** and **TPA-TT-TPD.**

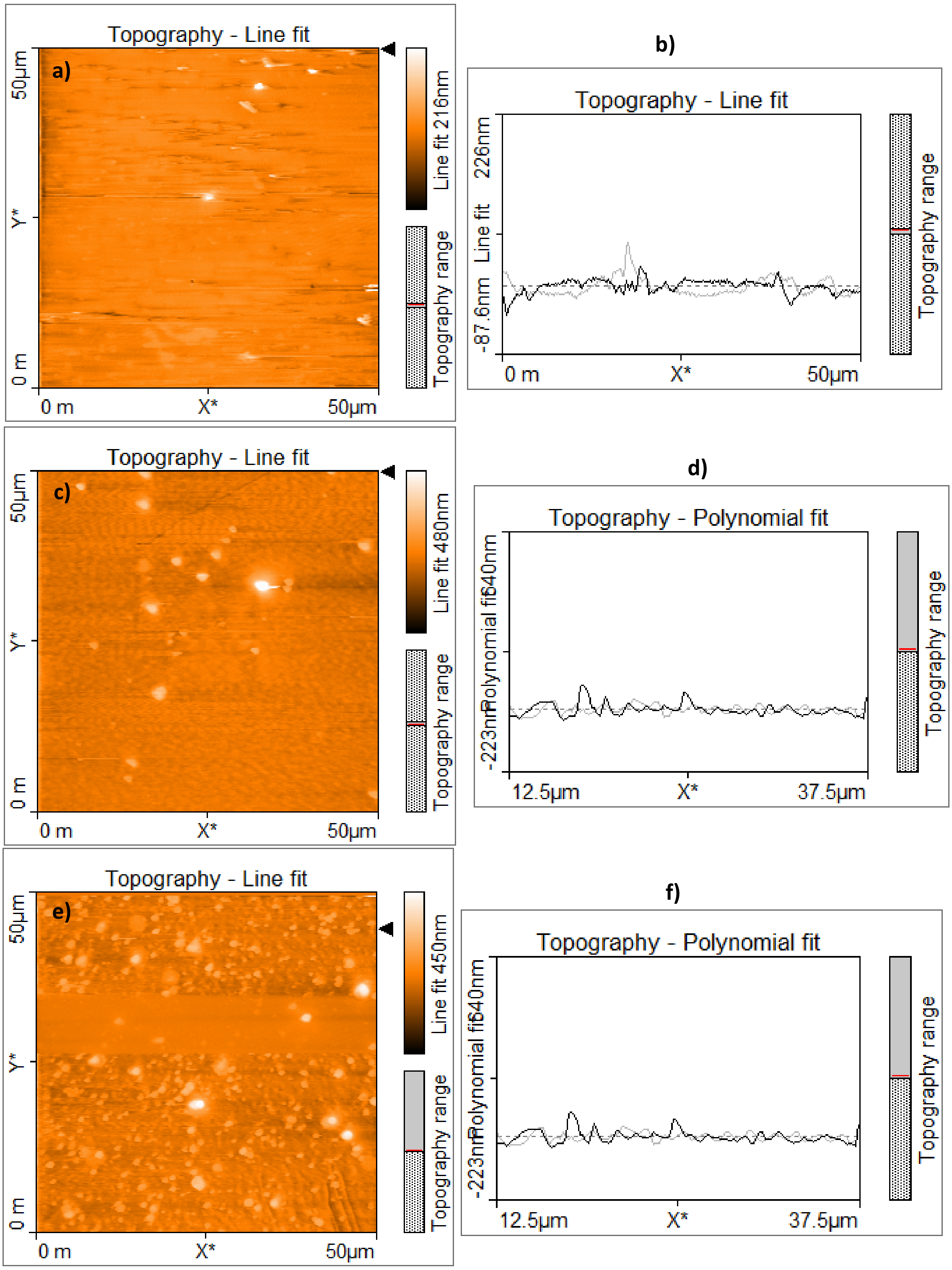

Figure S12. a), c), and e) correspond to AFM images of the active layers SM:$PC_{71}BM$ of **TPA-TPA**, **TPA-PT-TPD** and **TPA-TT-TPD** respectively. b), d), and f) correspond to root-mean-squared of the active layers SM:$PC_{71}BM$ of **TPA-TPA**, **TPA-PT-TPD** and **TPA-TT-TPD.**

7. References

[1] Zhan C. G.; Nichols J. A.; Dixon D. A. Ionization Potential, Electron Affinity, Electronegativity, Hardness, and Electron Excitation Energy: Molecular Properties from Density Functional Theory Orbital Energies. *J. Phys. Chem. A*. **2003**, *107*, 4184-4195.

[2] Romero-Borja, D.; Maldonado, J.L.; Barbosa-García, O.; Rodríguez, M.; Pérez-Gutiérrez, E.; Fuentes-Ramírez, R. G. Polymer Solar Cells Based on P3HT:$PC_{71}BM$ Doped at Different Concentrations of Isocyanate-Treated Graphene. *Synth. Met.* **2015**, 200, 91– 98

[3] Pérez-Gutiérrez, E.; Lozano, J.; Gaspar-Tánori, J.; Maldonado, J. L.: Gómez, B.; López, L.; Amores-Tapia L. F.; Barbosa-García, O.; Percino, M. J.; Organic Solar Cells all Made by Blade and Slot–Die Coating Techniques, *Sol. Energy*. **2017**, *146*, 79–84.

[4] Álvarez-Fernández, A.; Maldonado, J.L.; Pérez-Gutiérrez, E.; Rodríguez, M.; Ramos-Ortíz, G.; Barbosa-García, O.; Meneses-Nava, M.A.; Zolotukhin,.M. G. Performance and Stability of PTB7:$PC_{71}BM$ Based Polymers Solar Cells, With ECZ and/or PVK Dopants, Under the Application of an External Electric Field. *J. Mater. Sci.-Mater.* **2016**, *27*, 6271–6281

[5] Barreiro-Argüelles, D.; Ramos-Ortiz, G.; Maldonado, J. L.; Pérez-Gutiérrez, E.; Romero-Borja, D.; Álvarez-Fernández, A. "PTB7:PC71BM-Based Solar Cells Fabricated With the Eutectic Alloy Field's Metal as an Alternative Cathode and the Influence of an Electron Extraction Layer. *IEEE J. Photovoltaics* **2017**, *7*, 191–198.

[6] Pérez-Gutiérrez E.; Barreiro-Argüelles D.; Maldonado J. L.; Meneses-Nava M. A.; Barbosa-García O.; Ramos-Ortíz G.; Rodríguez M.; Fuentes-Hernández C., Semiconductor Polymer/Top Electrode Interface Generated by Two Deposition Methods and Its Influence on Organic Solar Cell Performance, *ACS Appl. Mater. Interfaces*, **2016**, *8*, 28763–28770

[7] Zhou, N.; Guo, X.; Ortiz, R. P.; Harschneck, T.; Manley, E. F.; Lou, S. J.; Hartnett, P. E.; Yu, X.; Horwitz, N. E.; Burrezo, P. M.; Aldrich, T. J.; Navarrete-López, J. T.; Wasielewski M. R.; Chen, L. X.; Chang, R. P. H.; Facchetti, A.; Marks, T. J. Marked Consequences of Systematic Oligothiophene Catenation in Thieno[3,4-c]pyrrole-4,6-dione and Bithiopheneimide Photovoltaic Copolymers. *J. Am. Chem. Soc.* **2015**, *137*, 12565−12579

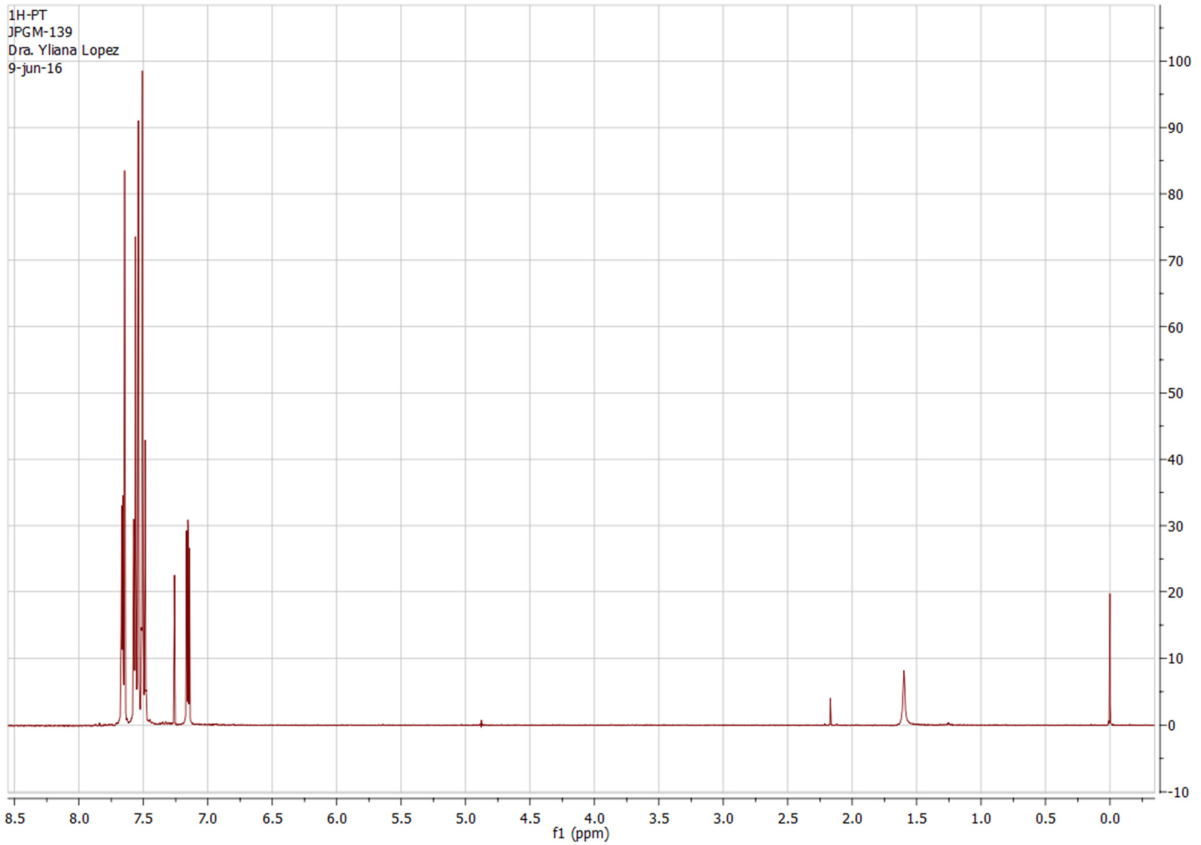

Figure S12. ^{1}H NMR for PT in $CDCl_3$ solution

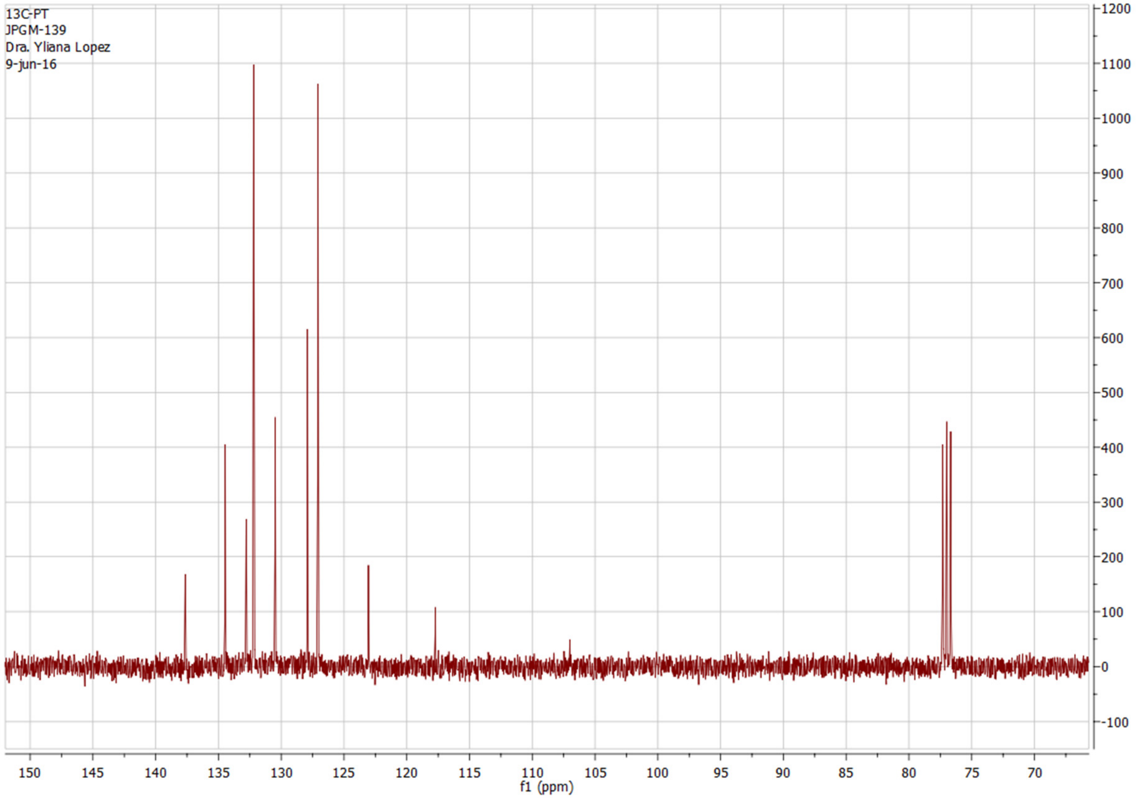

Figure S13. ^{13}C NMR for PT in $CDCl_3$ solution

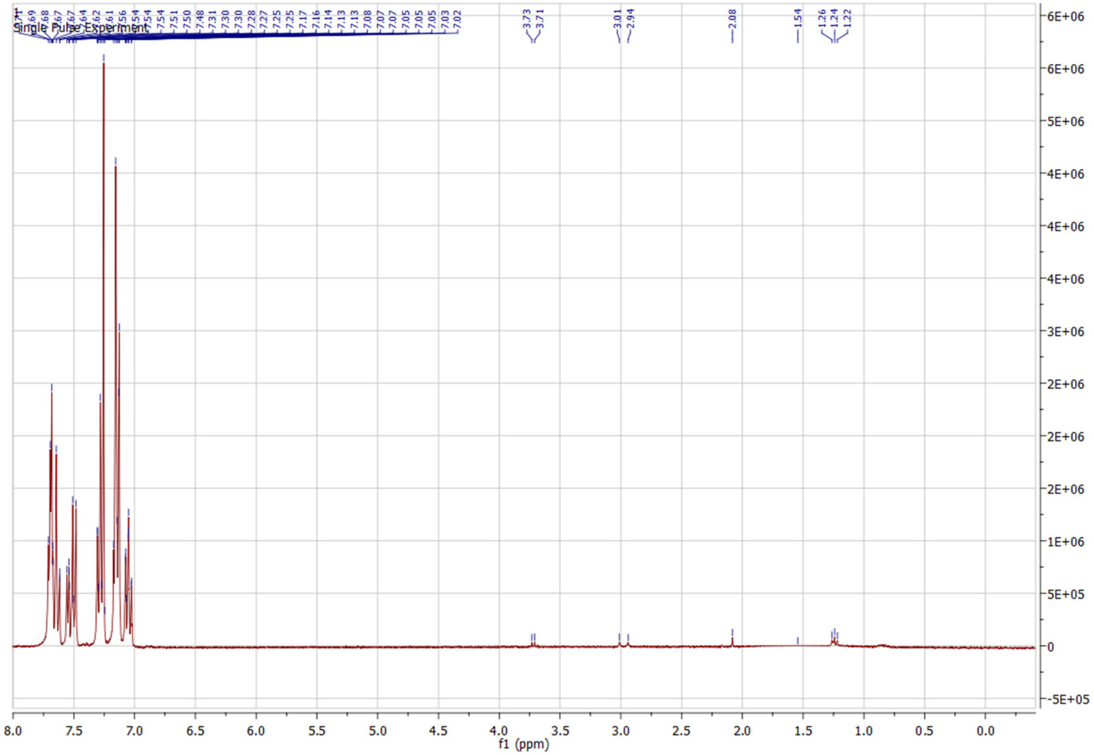

Figure S14. ^{1}H NMR for TPA-PT in $CDCl_3$ solution

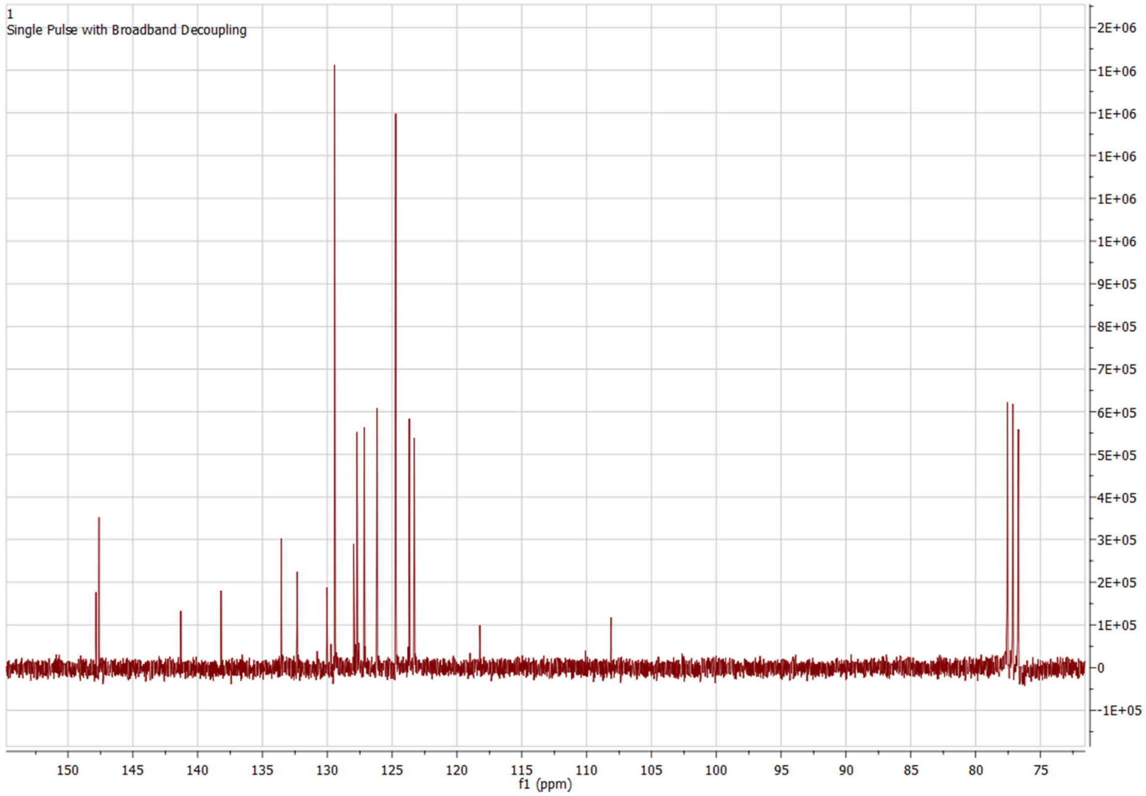

Figure S15. ^{13}C NMR for TPA-PT in $CDCl_3$ solution
